# Cryptochromes Define a Novel Circadian Clock Mechanism in Monarch Butterflies That May Underlie Sun Compass Navigation

**DOI:** 10.1371/journal.pbio.0060004

**Published:** 2008-01-08

**Authors:** Haisun Zhu, Ivo Sauman, Quan Yuan, Amy Casselman, Myai Emery-Le, Patrick Emery, Steven M Reppert

**Affiliations:** 1 Department of Neurobiology, University of Massachusetts Medical School, Worcester, Massachusetts, United States of America; 2 Biology Center, Institute of Entomology, Czech Academy of Sciences, Ceske Budejovice, Czech Republic; Howard Hughes Medical Institute, United States of America

## Abstract

The circadian clock plays a vital role in monarch butterfly (Danaus plexippus) migration by providing the timing component of time-compensated sun compass orientation, a process that is important for successful navigation. We therefore evaluated the monarch clockwork by focusing on the functions of a *Drosophila*-like *cryptochrome* (*cry*), designated *cry1*, and a vertebrate-like *cry*, designated *cry2*, that are both expressed in the butterfly and by placing these genes in the context of other relevant clock genes in vivo. We found that similar temporal patterns of clock gene expression and protein levels occur in the heads, as occur in DpN1 cells, of a monarch cell line that contains a light-driven clock. CRY1 mediates TIMELESS degradation by light in DpN1 cells, and a light-induced TIMELESS decrease occurs in putative clock cells in the pars lateralis (PL) in the brain. Moreover, monarch *cry1* transgenes partially rescue both biochemical and behavioral light-input defects in *cry^b^* mutant *Drosophila*. CRY2 is the major transcriptional repressor of CLOCK:CYCLE-mediated transcription in DpN1 cells, and endogenous CRY2 potently inhibits transcription without involvement of PERIOD. CRY2 is co-localized with clock proteins in the PL, and there it translocates to the nucleus at the appropriate time for transcriptional repression. We also discovered CRY2-positive neural projections that oscillate in the central complex. The results define a novel, CRY-centric clock mechanism in the monarch in which CRY1 likely functions as a blue-light photoreceptor for entrainment, whereas CRY2 functions within the clockwork as the transcriptional repressor of a negative transcriptional feedback loop. Our data further suggest that CRY2 may have a dual role in the monarch butterfly's brain—as a core clock element and as an output that regulates circadian activity in the central complex, the likely site of the sun compass.

## Author Summary

During their spectacular fall migration, eastern North American monarch butterflies (Danaus plexippus) use a time-compensated sun compass to help them navigate to their overwintering sites in central Mexico. The circadian clock plays a critical role in monarch butterfly migration by providing the timing component to time-compensated sun compass orientation. Here we characterize a novel molecular clock mechanism in monarchs by focusing on the functions of two CRYPTOCHROME (CRY) proteins. In the monarch clock, CRY1, a *Drosophila*-like protein, functions as a blue-light photoreceptor for photic entrainment, whereas CRY2, a vertebrate-like protein, functions within the clockwork as the major transcriptional repressor of the self-sustaining feedback loop. An oscillating CRY2-positive neural pathway was also discovered in the monarch brain that may communicate circadian information directly from the circadian clock to the central complex, which is the likely site of the sun compass. The monarch clock may be the prototype of a clock mechanism shared by other invertebrates that express both CRY proteins, and its elucidation will help crack the code of sun compass orientation.

## Abstract 3

An ancestral circadian clock mechanism is defined in monarch butterflies, in which two CRYPTOCHROME proteins function as critical components. The proteins may also function as output molecules that connect the clock to the sun compass for successful navigation.

## Introduction

In insects, circadian clocks regulate the timing of numerous biological events [1]. Some examples of critical circadian rhythm outputs in holometabolous insects include the time of day of adult eclosion, the seasonal timing of reproductive diapause, and time-compensated sun compass navigation.

The molecular clock mechanism has been the subject of intense investigation in *Drosophila* [2,3], while less attention has been directed at the clockwork mechanism in other, non-drosophilid insects. In the fruit fly, the central clock is driven primarily by a negative transcriptional feedback loop that involves the products of the *period* (*per*), and *timeless* (*tim*) genes, and the transcription factors *Clock* (*Clk*) and *cycle* (*cyc*). CLK and CYC heterodimers drive *per* and *tim* transcription through E-box enhancer elements. The resultant PER and TIM proteins form heterodimers that translocate back into the nucleus to repress their own transcription via inhibitory effects on CLK and CYC. *Drosophila* CRYPTOCHROME (CRY) is co-localized in clock cells with PER and TIM and functions as a blue-light photoreceptor involved in photic entrainment [4–6]. CRY disrupts PER and TIM heterodimers by directly interacting with TIM in a light-dependent process [7–9], and it also participates in its own light-dependent degradation [10].

The eastern North American monarch butterfly (Danaus plexippus) is well known for its long-distance fall migration [11]. We have been developing this species as a model to examine the role of the circadian clock in time-compensated sun compass orientation and in the seasonal induction of the migratory generation [12]. Using clock protein expression patterns, we previously identified the location of circadian clock cells in the dorsolateral protocerebrum (pars lateralis [PL]) of the butterfly [13], which expresses PER, TIM, and a *Drosophila*-like CRY (designated CRY1; see below). We also identified a CRY1-staining neural pathway that may connect the circadian (navigational) clock to polarized light input entering brain, which is important for sun compass navigation [14,15]. A CRY1 pathway also may connect the circadian clock to neurosecretory cells in the pars intercerebralis (PI) for the initiation of the migratory state [12,13]. A direct clock-to–sun compass pathway has also been postulated [13].

In the course of our molecular investigations of the circadian clock mechanism in monarchs, we have discovered that these butterflies, like all other non-drosophilid insects so far examined, express a second *cry* gene that encodes a vertebrate-like protein designated insect CRY2 [16]. Functional studies in *Drosophila* Schneider 2 (S2) cells show that monarch CRY2 is light insensitive, but potently inhibits CLOCK:CYCLE-mediated transcription, whereas monarch CRY1 is light sensitive, but does not show transcriptional repressive activity. However, the mechanistic details of CRY2′s actual function within a clockwork have not been defined in any insect.

Further molecular evolutionary studies have shown that gene duplication and loss have led to three modes of *cry* gene expression in insects, giving rise to three types of circadian clocks [17]: two derived clocks, in which only *cry1* (e.g., *Drosophila*) or *cry2* (e.g., the honey bee Apis mellifera and red flour beetle Tribolium castaneum) is expressed, and an ancestral clock in which both *cry1* and *cry2* are expressed (e.g., the monarch butterfly). The expression of two functionally distinct *cry*s in monarchs suggests that the butterfly clock may use a novel clockwork mechanism that is not yet fully described in any organism.

In the studies discussed here, we have therefore used in vivo approaches, a monarch cell line that contains a light-driven molecular clock, and *Drosophila* carrying monarch *cry1* or *cry2* transgenes to elucidate the monarch clockwork mechanism and its photic entrainment. Our results define many characteristics of a CRY-centric clock in the monarch butterfly with CRY1 functioning potentially as a blue-light photoreceptor for photic entrainment, whereas CRY2 functions, without PER, within the clockwork as the major transcriptional repressor of the core transcriptional feedback loop. We also present evidence of a CRY2-positive neural pathway that oscillates in the central complex, the apparent site of the sun compass [18,19]. CRY2 may thus function as both a core clock element and as an output-regulating circadian activity in the central complex.

## Results/Discussion

### Temporal Patterns of Clock Gene RNA and Protein Expression in Monarch Heads

If a negative transcriptional feedback loop underlies the circadian clock in monarch butterflies, it should drive the rhythmic expression of *per* and *tim* in vivo. We thus used quantitative real-time polymerase chain reactions (qPCRs) to examine the temporal expression patterns of the clock gene homologs in monarch butterfly heads at 3-h intervals in a 12 h light:12 h dark cycle (LD) and during the first day in constant darkness (DD).

**Figure 1:**
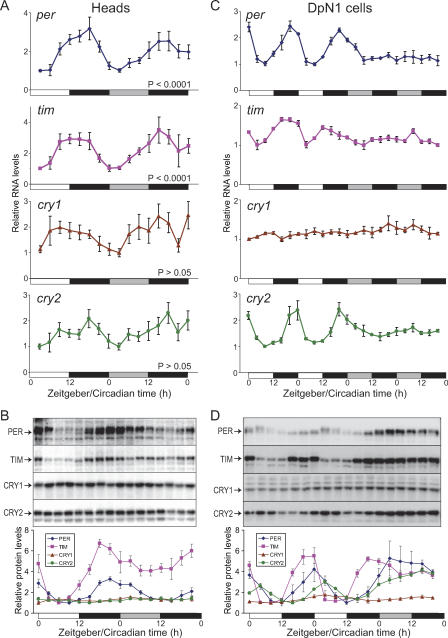
Temporal Patterns of Clock Gene RNA and Protein Expression in Monarch Heads and DpN1 Cells (A) Temporal profiles of clock gene RNA expression in heads. Heads were collected at 3-h intervals for 24 h in LD and during the first day in DD. RNA levels were quantitated by qPCR. Each value is the mean ± SEM from 6 sets of heads. Open bars, light; black bars, dark; gray bars, subjective day. *p-*value determined by one-way ANOVA. (B) Temporal profiles of clock proteins in heads. Heads were collected at 3-h intervals for 24 h in LD and during the first day in DD. Extracts were prepared, analyzed by Western blot and probed for PER (GP40), TIM (GP47), CRY1 (GP37), and CRY2 (GP51). Blots were imaged by chemiluminescence, and band intensity was quantified. The results were normalized against α-tubulin. Each value is the mean ± SEM from six heads. In LD: PER and TIM, *p <* 0.001. In DD: PER, *p* < 0.01; TIM, *p >* 0.05. (C) Temporal profiles of clock gene RNA expression in DpN1 cells. Cells were collected at 4-h intervals for two days in LD followed by two days in DD, and RNA levels were quantitated by qPCR . Each value is the mean ± SEM of three collections. (D) Temporal profiles of clock proteins in DpN1 cells. Cell homogenates were prepared, analyzed by Western blot and probed for PER (GP40), TIM (GP47), CRY1 (GP37), and CRY2 (GP51). Blots were imaged by chemiluminescence, and the band intensity was quantified. The results were normalized against α-tubulin. Each value is the mean ± SEM of three collections. In LD, PER, *p <* 0.05; for TIM and CRY2, *p <* 0.001.

Monarch *per* RNA levels exhibited a daily rhythm in LD with peak levels at Zeitgeber time (ZT) 18 and low levels at ZT 0–3, and the rhythm persisted in DD (*p* < 0.0001, one-way analysis of variance [ANOVA]) (Figure 1A), as previously described [20]. We found that a rhythm of similar phase was manifested by monarch *tim* RNA levels in both LD and DD (*p* < 0.0001) (Figure 1A). We also examined *cry1* and *cry2* RNA levels; although each RNA profile showed a similar trend, neither exhibited a significant daily rhythm (*p* > 0.05) (Figure 1A).

Monarch-specific antibodies against PER, TIM, CRY1, and CRY2 were used to examine the temporal profiles of clock protein abundance in monarch head extracts by Western blot analysis. Indeed, PER and TIM showed significant temporal oscillations in abundance in LD (*p* < 0.001), with peak levels occurring from ZT 18–24/0 (Figure 1B). There were also temporal changes in PER electrophoretic mobility; the changes in mobility were due to changes in phosphorylation, as phosphatase treatment converted >90% of the high–molecular weight forms of PER to a single, lower–molecular weight band (Figure S1). The more highly phosphorylated forms of PER were predominant at 3 h after lights-on. In DD, the oscillation in PER abundance persisted (*p* < 0.01), while the oscillation in TIM abundance was markedly blunted, to the point that there was no longer a significant daily rhythm (*p* > 0.05). Thus, the daily TIM abundance oscillation in the head is mainly light driven. There was no significant daily change in either monarch CRY1 or CRY2 abundance in whole head extracts in either LD or DD (*p* > 0.05) (Figure 1B).

### DpN1 Cells: A Monarch Cell Line with a Light-Driven Clock

We evaluated a monarch butterfly cell line designated DpN1 [21], which was originally derived from embryos, for expression of circadian clock RNAs and proteins, because such a cell line might be useful for helping us delineate the molecular clock mechanism in the butterfly. In DpN1 cells, we in fact found that the RNAs for *per*, *tim*, *cry1*, *cry2*, *Clk*, *cyc*, *vrille*, *Pdp1*, *slimb*, *doubletime*, *CKII*α, *CKII*β, and *shaggy* were all expressed (see Table S1). We focused our studies of the temporal dynamics of clock gene expression in DpN1 cells on *per*, *tim*, *cry1*, and *cry2* to parallel our in vivo analyses.

Remarkably, when studied at 4-h intervals under LD, we found cycling in clock gene RNA levels (by qPCR) and clock protein abundance (by Western blot analysis). At the level of gene expression, we found that monarch *per*, *tim*, and *cry2* exhibited near-synchronous daily rhythms in RNA levels, with peak levels between ZT 16 and 24, and trough levels between ZT 4 and 8 (*p* < 0.001) (Figure 1C). There was no significant daily oscillation in *cry1* levels in LD (*p* > 0.05). In DD, no clock gene RNA oscillation was apparent on the first day. This lack of a circadian oscillation was consistently observed in repeated experiments.

At the protein level, monarch PER and TIM showed robust temporal oscillations in abundance in DpN1 cells in LD, with highest protein levels at the end of the dark period (ZT 24/0) (*p* < 0.05 for PER and *p* < 0.001 for TIM; Figure 1D). CRY2 also showed temporal changes in abundance, with highest levels 4 h later at ZT 4 (*p* < 0.001; Figure 1D). For PER, there was not only a diurnal change in protein abundance but also in electrophoretic mobility, as found in head extracts (Figure S1). Phosphorylated PER was the dominant form at ZT 4, which correlated with the highest level of CRY2 abundance, and the rapidly declining *per*, *tim*, and *cry2* RNA levels. This temporal increase in CRY2 abundance in DpN1 cells contrasts with the lack of rhythmicity in CRY2 abundance over the 24-h day in LD in monarch heads (compare Figure 1B with 1D). The reason for this discrepancy is because CRY2 is more widely expressed in the monarch brain than the other clock proteins examined, and CRY2 is not under robust circadian control in most areas (see below). The temporal profiles of clock gene RNA and protein expression in DpN1 cells are consistent with PER and/or CRY2 being involved in negative feedback repression of CLK:CYC-mediated transcription in the cell line, which is further explored below. Similar to what we found for RNA expression in DpN1 cells, we were unable to identify a circadian oscillation of the clock proteins in the cells in DD (Figure 1D).

Although it is unclear why we were not able to detect a functional circadian clock in DpN1 cells, the close correlation of clock gene RNA and protein expression patterns between DpN1 cells and heads in LD, makes the cell line a useful system in which to study the molecular and biochemical details of the monarch clock transcriptional feedback loop in LD (focusing on the role of CRY2), as well as its intracellular light input pathway (focusing on the role of CRY1).

### CRY1 Mediates the Light-Induced Decrease in TIM in DpN1 Cells

**Figure 2:**
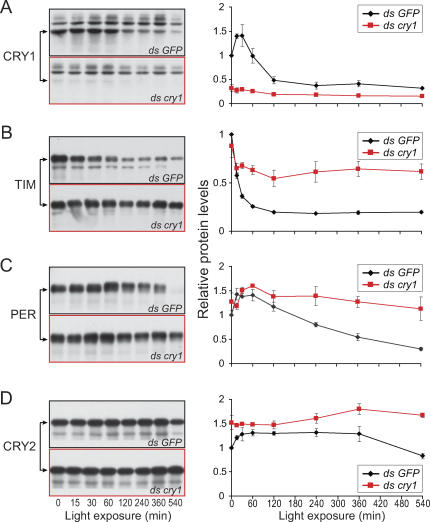
CRY1 and TIM Reponses to Light in DpN1 Cells Clock protein abundance in LD-cultured cells changes in response to light. DpN1 cells were cultured under LD, pretreated with dsRNA against *GFP* (black lines) or dsRNA against *cry1* (red lines), and then exposed to light (at the start of the normal light period) for 540 min. Cells were collected at the designated times. Cell homogenates were analyzed by Western blot, and probed for CRY1 (GP37), TIM (GP47), PER (GP40), and CRY2 (GP51) (left-hand panels). The time courses of declines were quantified by chemiluminescence, and band intensity was normalized against α-tubulin (right-hand panels). (A) CRY1, (B) TIM, (C) PER, (D) CRY2. Time 0 is before lights on. Each point is the mean ± SEM of three experiments.

We first used DpN1 cells to examine whether monarch CRY1 mediates the light-induced decrease in TIM abundance, providing a light-resetting pathway into the molecular clock. By using RNA interference induced by double-stranded RNAs (dsRNAs,) we supply evidence that the light-induced decrease in TIM abundance in DpN1 cells is mediated through CRY1 (Figure 2 and Figure S2).

Once lights were turned on to initiate the normal light period in LD-cultured control cells (those treated with double-stranded RNA [dsRNA] targeting the green fluorescent protein [*GFP*] gene), there was a transient increase in CRY1 abundance at 15 and 30 min (Figure 2A, black line), followed by a rapid decrease by 60 min, reaching constant low levels by 120 min; the light-induced decrease in CRY1 abundance in LD-cultured cells was unexpected (see below). With lights on, there was a rapid decrease in TIM abundance at 15 min, reaching constant low levels by 60 min (Figure 2B, black lines). Light induced a slower decrease in PER abundance starting at 120 min, with a steady decline throughout the light period (Figure 2C, black line). The light-induced decline in CRY2 abundance was even slower and only apparent at 540 min (Figure 2D, black line). The time course of light-induced protein decrements from TIM to CRY2 was similar to that seen after lights on (ZT 12) in LD without dsRNA treatment (Figure 1D) and is consistent with a series of protective protein:protein interactions in which TIM:PER interactions protect PER from degradation, whereas PER:CRY2 interactions protect CRY2 from degradation (see below).

A surprising aspect of the control experiment was that the initiation of the light period now caused a decrease in CRY1 abundance in cells treated with dsRNA targeting *GFP*, rather than CRY1 levels remaining at constant dark-like levels in the light, as seen in untreated cells cultured under LD (Figure 1D). This light-induced CRY1 decrease was found to be secondary to a 5-h serum starvation of the medium that is necessary for efficient transfection of dsRNA into DpN1 cells (unpublished data); serum starvation likely induces the expression of a kinase that is important for monarch CRY1′s proteasomal degradation by light (see Figure S2).

Nonetheless, pretreatment of cells maintained in LD with dsRNA targeting *cry1*, which caused a ∼60% reduction in CRY1 abundance in darkness just prior to (time 0) and throughout light exposure (Figure2A, red line), greatly reduced the decrease in TIM abundance in response to light (Figure 2B, red lines). Pretreatment also greatly reduced the subsequent decreases in PER and CRY2 abundance (Figure 2C and 2D, red lines), compared with controls (cells treated with dsRNA targeting *GFP*). The lack of a complete block of the light-induced reduction of TIM appeared to be secondary to the partial CRY1 knockdown (see Figure 2A). The dsRNA data strongly suggest that CRY1 mediates the light-induced TIM degradation in DpN1 cells (see also Figure S2).

Consistent with CRY1-mediating photic entrainment in the butterfly [22], we found that blue light is the spectral component that degrades CRY1 and TIM in DpN1 cells and also synchronizes the timing of behavior (the adult eclosion rhythm) to the 24-h day (Figure S3).

### TIM Localization and Light Sensitivity in the Brain

**Figure 3:**
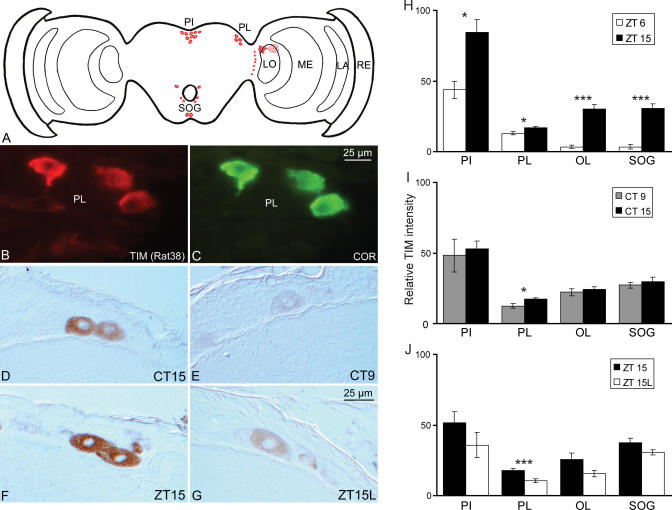
Distribution and Regulation of TIM Immunoreactivity in Monarch Brain (A) Schematic representation of a frontal section illustrating the topology of TIM-immunoreactive cells using antibody TIM-R38. Although an identical pattern of TIM staining was obtained with TIM-GP47, TIM-R38 was used in experiments depicted in (B–J), because of stronger signal intensity. RE, retina; LA, lamina; ME, medulla; LO, lobula of optic lobe (OL); PL, pars lateralis; PI pars intercerebralis; SOG, suboesphageal ganglion. (B and C) Double-labeling immunofluorscence of TIM (B) and corazonin (COR; C) in cells in the PL. The three cells shown are co-localized with TIM and COR; the fourth cell was out of the plane of section. (D and E) TIM staining in PL at CT 15 (D) and CT 9 (E). Two cells are shown; the other two were out of the plane of section. (F and G) TIM staining in PL at ZT 15 in darkness (F) or after a 1-h light pulse (ZT 15L) (G). Two cells are shown; the other two were out of the plane of section. (H) Semiquantitative assessment of TIM staining in PI, PL, OL, and SOG at ZT 6 and ZT 15. Intensity values were corrected for relative cell number in each group so that the values could be compared across groups. Each value is mean ± SEM of four animals. *p < 0.05; ***p < 0.001. (I) Semiquantitative assessment of TIM staining in PI, PL, OL, and SOG at the two circadian times (CT 9 and CT 15). Each value is mean ± SEM of eight animals. *p < 0.05. (J) Semiquantitative assessment of TIM staining in PI, PL, OL, and SOG before and after the light pulse (ZT 15 and ZT 15L, respectively). Each value is mean ± SEM of eight animals. ***p < 0.001.

Next, we examined the location of light-sensing clock cells in monarch brain by immunocytochemistry, using our newly-developed monarch-specific anti-TIM antibodies. Monarch TIM-like immunoreactivity was detected by the new antibodies in the cytoplasm of cells in the PI and PL (Figure 3A–3G and unpublished data), as previously described using an anti-TIM antibody against *Drosophila* TIM [13]. Each of the monarch-specific antibodies gave prominent staining patterns in the cytoplasm (compared with weak staining with the *Drosophila* antibody, see [13]), with ∼25 large cells stained in the PI and four cells consistently stained in the PL. In addition, approximately eight cells were identified near the lobula region of the optic lobe (OL), and approximately eight cells were found in the suboesophageal ganglion (SOG). Double-labeling studies showed that the cytoplasmic TIM staining was localized in the PL to the four cells that co-express corazonin (Figure 3B and 3C), a neuropeptide that marks clock cells in the PL of lepidopteran brains [23,24], including monarchs, in which two of the four cells also stain for PER and CRY1 [13]. Moreover, direct comparison confirmed that the cytoplasmic staining of CRY1 and TIM were colocalized in two of the four cells in PL (Figure S4). We were unable to determine whether CRY1 and TIM were colocalized in the PI, however, because of weak staining for CRY1 in this structure; because there were twice as many TIM-positive cells as CRY1-positive cells in PI, only half of those TIM-positive cells would be expected to be colocalized with CRY1. The anti-TIM antibodies also stained a group of cells in the dorsal region of the OL (Figure 3A and Figure S5) in close vicinity, but not identical to the CRY1-positive group of cells previously described there [13]. These TIM-positive cells projected into the same glomerular structure as the adjacent CRY1-staining cells (Figure S5). We did not observe detectable TIM staining in the nuclei of any of the cell groups.

All of the cyptoplasmic staining in TIM-positive cells in the brain appeared to be light sensitive in LD. As previously noted, Western blot data showed a large light-driven daily oscillation of TIM in heads under LD conditions, with the daily oscillation of TIM abundance substantially blunted on placement in DD (Figure 1B). A similar pattern was found for the TIM-positive cells in the brain. In LD, all TIM-positive regions exhibited significantly lower levels of TIM staining at ZT 6, compared to ZT 15, including all four TIM-positive cells in PL (Figure 3H). In DD, on the other hand, there was a significant oscillation in PL only (*p <* 0.05), with lower staining at circadian time (CT) 9 and higher staining at CT 15 (Figure 3D, 3E, and 3I). In all other areas (PL, OL, and SOG), there was no significant difference between CT 9 and CT 15 (*p >* 0.05). When subjected to a 1-h light pulse from ZT 14–15 (ZT 15L), a significant light-induced decrease in TIM levels was detected in the PL only (*p <* 0.01), affecting all four TIM-positive cells, compared with brains kept in the dark (Figure 3F, 3G, and J). In all other areas (PI, OL, and SOG), there was a clear trend for a decrease in TIM staining with the light pulse (Figure 3J), but it did not reach significance (*p >* 0.05). Collectively, the data show that there is a good correlation in the different lighting schedules between TIM abundance changes in heads detected by Western blots and TIM staining patterns in brain regions detected by immunocytochemistry. TIM staining in the PL was the area most consistently regulated (by light and in DD).

These data show a complex relationship between CRY1 and TIM degradation in the monarch brain. Wherever CRY1 and TIM are colocalized, CRY1 likely mediates TIM degradation, based on our studies in DpN1 cells (Figure 2). In the other TIM-positive areas, either CRY1 is present below the level of antibody detection or TIM in those cells is degraded in a CRY1-independent manner, perhaps by local interactions (as may occur in PL), by opsins expressed in brain, and/or by neural pathways from eye and/or stemmata to TIM-positive cells.

We showed previously by immunocytochemistry that CRY1 levels in the PI and PL are not altered by light exposure [13]. It thus appears that the light-induced decrease in TIM in TIM/CRY1 colocalized cells is not necessarily accompanied by a measurable decrease in monarch CRY1 abundance, which has also been shown by Western blot analysis in LD (Figure 1B and 1D) and with short-term light exposure at night both in DpN1 cells and in whole-head extracts (Figure S6), as well as in *Drosophila* [7]. It thus appears that light may induce a conformational change in monarch CRY1, leading to TIM degradation, but without necessarily inducing its own degradation.

### A Monarch *cry1* Transgene Partially Rescues Light-Input Defects in *cry^b^ Drosophila*

Because there are no genetic approaches yet available in monarch butterflies [12], we asked whether monarch CRY1 can function as a circadian photoreceptor by expressing monarch transgenes in *Drosophila*. We used the GAL4-UAS system, with *tim*-GAL4 as the driver, which drives transgene expression in clock neurons that generate the circadian locomotor activity rhythm [25]. For these studies, we took advantage of the *cry^b^* mutation in *Drosophila*, because it induces severe light-input defects; circadian phase does not shift in response to a light pulse, and TIM does not cycle in LD [4–6]. We attempted to rescue these phenotypes by expressing *UAS-monarch cry1* or *UAS-monarch cry2* transgenes in the *cry^b^* background.

**Figure 4:**
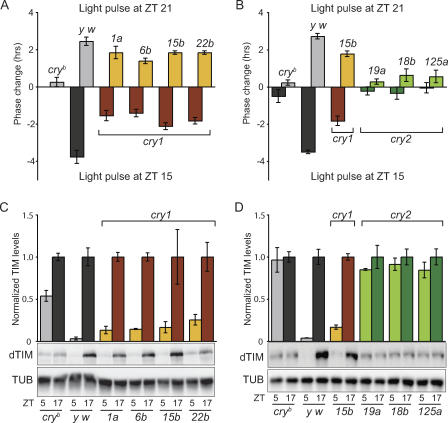
Transgene Expression of Monarch *cry1* Partially Rescues *cry^b^* Defects (A) Expression of monarch CRY1 in a *cry^b^* background partially rescues phase advances and delays after a 1-h light pulse at ZT 21 or ZT 15, respectively. Four independent *UAS-cry1* lines—designated *1a*, *6b*, *15b*, and *22b*—were examined. Expression of the transgenes was driven by *tim-GAL4*. Phase changes: positive numbers are advances, negative numbers are delays. The ZT 21 pulse experiment was performed three times for *cry^b^*, *y w*, *6b*, *15b*, and *22b*, and twice for *1a*, using 16 males per genotype per experiment. The ZT 15 pulse experiment was performed four times for *cry^b^*, *y w*, *6b*, *15b*, and *22b*, and three times for *1a*, using 16 males per genotype per experiment. Each value is the mean ± SEM. The value for *cry^b^* at ZT 15 was 0 with SEM within the width of the horizontal line. (B) Expression of monarch CRY2 in a *cry^b^* background does not rescue phase shifts after a 1-h light pulse at either ZT 21 or ZT 15. Three independent *UAS-cry2* lines, designated *19a*, *18b*, and *125a*, were examined*.* The *UAS-cry1* line, *15b*, was included as a comparison. Expression of all the transgenes was driven by *tim-GAL4*. The ZT 21 and ZT 15 pulses experiments were performed three times each, using 16 males per genotype per light pulse per experiment. Each value is the mean ± SEM. (C) Expression of monarch CRY1 partially rescues *Drosophila* TIM cycling in LD. Flies were collected at ZT 5 and ZT 17. Whole head extracts were subjected to Western blot analysis using a *Drosophila* anti-TIM antibody (top half of blot) or anti-tubulin antibody (bottom half of same blot). The *UAS-cry1* lines are the same as in (A). TIM levels at ZT 17 were normalized to 1.0. This experiment was performed three times. Each value is the mean ± SEM. (D) Expression of monarch CRY2 does not rescue *Drosophila* TIM cycling in LD. The *UAS-cry2* lines are the same as in (B). The *UAS-cry1* line, *15b*, is included for comparison. TIM levels at ZT 17 are normalized to 1.0. This experiment was performed three times. Each value is the mean ± SEM.

We first examined the ability of the monarch *cry1* transgene to restore the ability of discrete light pulses at night to phase-shift the circadian clock that drives locomotor activity in *cry^b^* mutant flies. We used two light pulses; a 1-h light pulse at ZT 15, which normally causes phase delays, or a 1-h light pulse at ZT 21, which normally causes phase advances [5]. The light-pulse experiments using four independent *UAS-cry1* lines showed a partial rescue of the *cry^b^* phenotype. With a light pulse at ZT 21, the phase advances in the *UAS-cry1* lines *1a*, *15b*, and *22b* were as robust as the *y w* control (no significant differences), and the phase advance of line *6b* was only slightly less than that of *y w* (*p <* 0.05) (Figure 4A). With a light pulse at ZT 15, the rescue was still evident, but not as robust; all four *UAS-cry1* lines had a statistically smaller phase change than *y w* (*p <* 0.001 for each), but they also had a statistically larger phase change than the *cry^b^* line (*p <* 0.01 for *1a* and *6b*; *p <* 0.001 for *15b* and *22b*) (Figure 4A). When the same phase shift experiment was performed with three *UAS-cry2* lines—*19a*, *18b*, and *125a*—at both ZT 15 and ZT 21, the phase changes were minimal and not significantly different from the *cry^b^* line without transgene expression (*p >* 0.05) (Figure 4B).

Next, the four *UAS-cry1* lines were examined for their ability to rescue the light-induced, CRY-dependent TIM oscillations in heads of the *cry^b^* background. In *cry^b^* flies, TIM levels do not cycle in LD. It is known that the light-induced TIM oscillation can be rescued by expressing *Drosophila* CRY under the *tim-GAL4* driver [5]. Each of the *UAS-Cry1* lines partially rescued TIM cycling in fly heads (Figure 4C). Note that although TIM does not normally degrade in *cry^b^* flies, some degree of cycling is occasionally observed, as seen in this set of experiments (Figure 4C, lanes 1 and 2). When TIM cycling was examined in the three *UAS-cry2* lines in LD, TIM cycling was not restored, indicating that monarch CRY2 cannot rescue this *cry^b^* defect (Figure 4D).

The results of these behavioral (light pulse) and biochemical (TIM degradation) experiments strongly suggest that monarch CRY1 can function as a circadian photoreceptor in *Drosophila*, whereas monarch CRY2 cannot.

### Monarch CRY2, but Not PER, Represses CLOCK:CYCLE–Mediated Transcription in DpN1 Cells

Having provided several lines of evidence suggesting that CRY1 functions as a photoreceptor for the butterfly clock, we next used DpN1 cells to construct the primary gear of the circadian clock, a negative transcriptional feedback loop, by examining the ability of monarch PER, TIM, CRY1, or CRY2 to inhibit monarch (dp)CLK:dpCYC–mediated transcription. Previous studies in S2 cells have shown that monarch CRY2 is a potent repressor of dpCLK:dpCYC–mediated transcription [16,17], but it has also been shown in S2 cells that both the *Drosophila* and Antheraea pernyi PER proteins alone potently repress *Drosophila* (d) CLK:dCYC–mediated transcription [26–29]. The DpN1 cell line was ideal for the current study because it allowed for the exogenously expressed monarch proteins to be examined in a homologous cell-based system. We used luciferase reporter gene assays with a reporter construct containing a tandem repeat of the proximal CACGTG E-box enhancer from the monarch *per* gene promoter [16,17].

**Figure 5:**
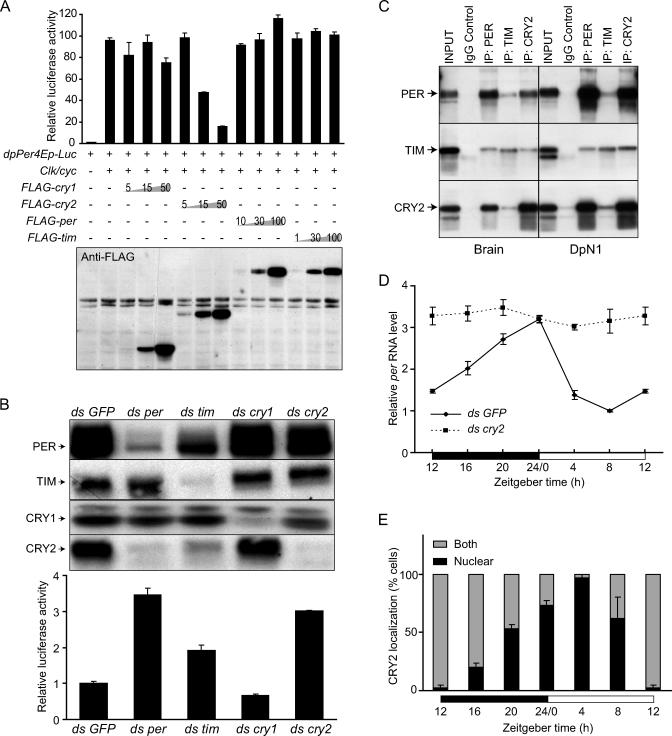
CRY2 Is a Major Repressor of CLK:CYC–Mediated Transcription in DpN1 Cells (A) Monarch CRY2 inhibits dpCLK:dpCYC–mediated transcription using luciferase reporter gene assays. The monarch butterfly *per* E box enhancer luciferase reporter (*dpPer4Ep-Luc*; 50 ng) was used in the presence (+) or absence (–) of monarch CLK/CYC expression plasmids (50 ng each). Monarch *cry1* (5, 15, and 50 ng), *cry2* (5, 15, and 50 ng), *per* (10, 30, and 100 ng), or *tim* (1, 30, and 100 ng) was used. Luciferase activity relative to β-galactosidase activity was computed. Each value is the mean ± SEM of three independent transfections. Western blot of FLAG-epitope–-tagged protein expression levels for each concentration of each construct is depicted below the graph. (B) De-repression assay showing that endogenous CRY2 inhibits dpCLK:dpCYC–mediated transcription. The monarch *per* E box luciferase reporter and monarch CLOCK and CYC were co-transfected into DpN1 cells to elevate reporter activity. The ability of endogenous PER, TIM, CRY1, or CRY2 to inhibit CLK:CYC-mediated transcriptional activity was then evaluated using dsRNA directed against each RNA to determine what effect knockdown had on the levels of all four clock proteins (Western blots using PER-GP40, TIM-GP47, CRY1-GP37, or CRY2-GP51, upper panel) and whether knockdown elevated (de-repressed) luciferase activity (lower panel). The luciferase values are the mean ± SEM of three independent experiments. (C) Monarch clock proteins form multimeric complexes in vivo. Brain or DpN1 extracts from ZT 18–19 were immunoprecipitated with antibodies against monarch PER (R33), TIM (R38), or CRY2 (R41). Immunocomplexes generated by each antibody were then analyzed by Western blot and probed for all three proteins (PER-GP40, TIM-GP47, and CRY2-GP51). (D) Knockdown of endogenous CRY2 abolishes the diurnal *per* RNA rhythm. dsRNA against monarch *cry2* was transfected into DpN1 cells to knock down CRY2 (Figure S9A), and *per* RNA levels were monitored at 4-h intervals over 24 h in LD. Double-stranded RNA against *GFP* served as the control. Relative monarch *per* RNA levels are depicted. Each value is mean ± SEM of three experiments. Solid line, *GFP* control; dashed line, CRY2 knockdown. (E) Oscillation in nuclear CRY2 abundance in DpN1 cells. Cells were entrained to LD and then fixed at 4-h intervals over 24 h in LD. The cellular localization of CRY2 was assayed by immunocytochemistry using CRY2-GP51. The cells were counterstained with SYTOX Blue to visualize the nuclei. At each time point, the localization of CRY2 in the cells was categorized as nuclear, cytoplasmic, or both nuclear and cytoplasmic. The proportion of cells at each time point in each category was calculated as the percentage of the total number of cells counted (30 per slide). Each bar represents the mean ± SEM of three experiments. Representative photomicrographs of CRY2 staining in nucleus, and in both cytoplasm and nucleus are shown in Figure S9B.

Cotransfection of the reporter with monarch CLK and CYC caused a 100-fold increase in transcriptional activity (Figure 5A). As expected, monarch CRY2 potently inhibited dpCLK:dpCYC–mediated transcription in a dose-dependent manner, yet neither monarch PER nor monarch TIM inhibited transcription (Figure 5A); transfected monarch PER is >90% nuclear in DpN1 cells (unpublished data). The same result was found with independent PER constructs obtained from cDNA from different sources of monarch head RNA (unpublished data). Monarch PER does have the potential to inhibit transcription in other cellular contexts, because it robustly inhibited dCLK:dCYC–mediated transcription in a dose-dependent manner in *Drosophila* S2 cells (unpublished data).

These data suggest that the monarch clock homologs can participate in a negative transcriptional feedback loop. A novel aspect of this feedback loop is that monarch CRY2 has the major inhibitory role for repressing dpCLK:dpCYC–mediated transcription from a monarch *per* E box enhancer, while PER was ineffective (either alone or in combination with TIM or sub maximal inhibitory doses of CRY2, Figure S7).

Next, a repressive effect of endogenous monarch CRY2 was examined on dpCLK:dpCYC–mediated transcription using dsRNAs to knock down endogenous clock gene expression in DpN1 cells. For one dsRNA approach, the monarch *per* E box luciferase reporter and monarch CLOCK and CYC were cotransfected to elevate reporter activity. The ability of endogenous PER, TIM, CRY1, or CRY2 to inhibit CLK:CYC–mediated transcriptional activity was then evaluated using dsRNA directed against each clock gene RNA to determine whether knockdown elevated (de-repressed) luciferase activity and what effect knockdown had on the levels of all four clock proteins.

The luciferase value obtained with dsRNA against *GFP* was the control for comparison of clock protein levels and knockdown-induced de-repression (Figure 5B, lane 1). Double-stranded RNA directed against *per* caused a substantial reduction in both PER and CRY2 abundance, and luciferase activity was elevated (de-repressed) by ∼3-fold (Figure 5B, lane 2). The decrease in CRY2 abundance with dsRNA against *per* did not appear to be the result of a decrease in *cry2* transcription (Figure S8), but was due to a post-transcriptional process, likely involving direct PER:CRY2 interactions, which protect CRY2 from degradation (see below). Double-stranded RNA against *tim* knocked down TIM abundance, and also caused a modest decrease in PER and CRY2 abundance, while luciferase reporter activity was elevated (de-repressed) 2-fold (Figure 5B, lane 3). Double-stranded RNA against *cry1* substantially reduced CRY1 abundance only, and did not cause an elevation in luciferase reporter activity compared to *GFP* control (Figure 5B, lane 4 versus lane 1). Double-stranded RNA against *cry2* caused a ∼70% reduction in CRY2 abundance only, while reporter activity was elevated (de-repressed) to a level comparable to the value with dsRNA against *per* (Figure 5B, lane 5 versus lane 2). Collectively, these data strongly suggest that endogenous CRY2 alone (not PER) is a dominant repressor of dpCLK:dpCYC–mediated transcription in DpN1 cells. The dsRNA knockdown results are also consistent with PER stabilizing CRY2 and TIM stabilizing PER (see also the temporal order of light-induced clock protein degradation, Figure 1D, Figure 2, and Figure S2A), and show that the de-repression following knockdown of PER or TIM is due to secondary reductions in CRY2 levels.

These biochemical data suggest that TIM, PER, and CRY2 are in the same protein complex. We therefore examined endogenous protein interactions by incubating DpN1 cell or brain extracts with clock protein antisera and probing the resulting immune complexes for each of the three clock proteins by Western blot analysis. Immunoprecipitated PER pulled down TIM and CRY2, immunoprecipitated TIM pulled down PER and CRY2, and immunoprecipitated CRY2 pulled down PER and TIM in both DpN1 cells and in brains (Figure 5C). These results are consistent with the existence of endogenous clock protein complexes containing PER, TIM, and CRY2. The data are also consistent with the protective protein interactions (TIM protects PER from degradation and PER protects CRY2 from degradation) suggested in previous experiments (see Figure 2, Figure S2A and Figure 5B).

In our second dsRNA approach, dsRNA against *cry2* was transfected into DpN1 cells to knock down CRY2, and *per* RNA levels were monitored at 4-h intervals over 24 h in LD, and dsRNA against *GFP* served as the control. We could not use dsRNA against *per* for this approach, because of the secondary effect of PER knockdown decreasing CRY2 levels, as documented above (Figure 5B, lanes 2). With *GFP* dsRNA, the normal daily oscillation of *per* RNA in LD was clearly apparent and unaltered with high levels from ZT 20–24 (Figure 5D). With CRY2 knockdown, on the other hand, *per* RNA levels remained at peak values throughout the 24-h period, with no oscillation (Figure 5D and Figure S9A). This result confirms that endogenous CRY2 is the major repressor of dpCLK:dpCYC–mediated transcription for this light-driven clock, because without substantial CRY2, *per* transcription remains constantly high over the 24-h period in LD. Moreover, the increase in PER levels with CRY2 knockdown again shows that endogenous CRY2 is the major repressor; there is no evidence for a role of PER in CRY2′s repressive ability in DpN1 cells.

If CRY2 is the transcriptional repressor of the diurnal clock in DpN1 cells, then its cellular localization should change over the day, being mainly nuclear at the time of maximal repression of dpCLK:dpCYC–mediated transcription. We thus examined the temporal profile of nuclear CRY2 in DpN1 cells and compared the time course to the normal daily rhythm in *per* RNA levels depicted in Figure 5D (solid lines), as a measure of dpCLK:dpCYC–mediated transcriptional readout. When the temporal profiles were examined at 4-h intervals over 24 h in LD, we found a clear daily change in the cellular location of CRY2 (Figure 5E and Figure S9B). The amount of CRY2 in the nucleus began to increase at ZT 16 and peaked at ZT 4, the predicted time of CRY2 maximal repression, when *per* RNA levels had dropped to near low values (Figure 5D). Because the low levels of *per* RNA persisted with increasing time in the light period of LD (ZT 8 and 12; Figure 5D), the amount of CRY2 in the nucleus began to decline (Figure 5E). These data show an oscillation in nuclear CRY2 abundance that is consistent with its role as the major transcriptional repressor of the light-driven clock in DpN1 cells. Perhaps in LD, only a portion of CRY2 in DpN1 cells—the portion translocated from cytoplasm to nucleus—is functionally relevant for inhibition of dpCLK:dpCYC–mediated transcription.

### Monarch CRY2 Is Co-Localized with Other Clock Proteins in the PL

But what about CRY2 function in the monarch brain? We first used in situ hybridization to map *cry2* RNA expression in the monarch brain. The brain distribution revealed RNA staining in ∼16 cells in the PI, four cells in the PL, ∼six cells in the central protocerebrum ventrally from the central body and dorsally from the oesophageal foramen, and ∼four cells in the SOG (Figure S10A–S10C). There was also extensive staining in the OLs, which included cells in the dorsal and ventral OL, and several hundred small cells that were found between the lobula and medulla, between the medulla and lamina, and between the lamina and retina (Figure S10A).

**Figure 6:**
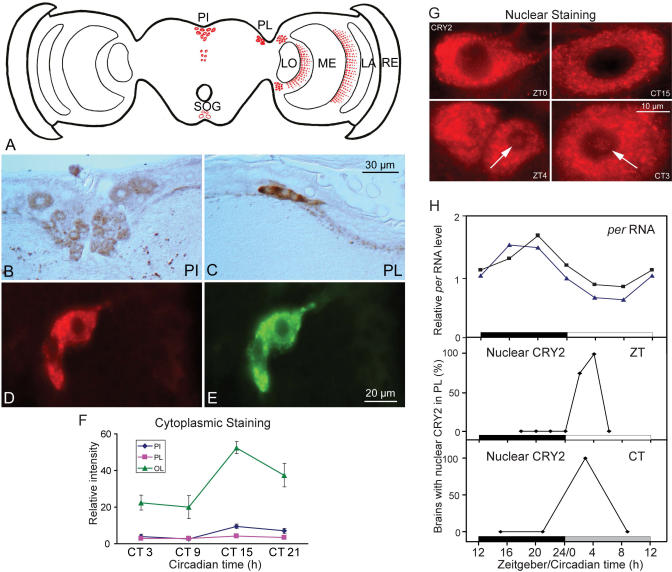
CRY2 Protein Distribution and Nuclear Localization in Monarch Brain. (A) Schematic representation of a frontal section illustrating the topology of CRY2-immunoreactive cells using antibody CRY2-R42. A similar pattern of CRY2 staining was found using CRY2-GP51 (see Figure S13). (B) CRY2 immunoreactivity in neurosecretory cells in the PI. (C) CRY2 immunoreactivity in cells in the PL. (D and E) Double-labeling immunofluorescence of CRY2 (D) and COR (E) in two cells in the PL. The other two co-localized cells were out of the plane of section. (F) Semiquantitative assessment of CRY2 immunostaining in the PI, PL, and dorsal and ventral OL on the first day in DD. Intensity values were corrected for relative cell number in each group so that the values could be compared across groups. Each point is mean ± SEM of 5–6 brains. For PI, *p <* 0.01, one-way ANOVA; PL, *p <* 0.05; OL, *p <* 0.01. (G) Nuclear localization of CRY2 using antibody to CRY2-R42. CRY2 staining in PL at ZT 0, top left; ZT 4, bottom left; CT 15, top right; and CT 3, bottom right. DAPI counterstaining was used to define the nucleus (not shown). CRY2 staining was not found in the nucleus at ZT 0 or CT 15, but it was found in the nucleus in PL at ZT 4 and CT 3 (arrows). (H) Comparison of *per* RNA levels in brain with temporal patterns of CRY2 nuclear staining in PL. Upper, *per* RNA levels for two sets of dissected brains without photoreceptors (black and blue lines) collected at 4-h intervals over 24 h in LD. Middle, nuclear CRY2 staining in PL at seven ZT times plotted as % of brains examined (*n* = 4–5 brains at each time point). Lower, nuclear CRY2 staining in the PL at four time points over the circadian cycle plotted as percent of brains examined (*n* = 4–5 brains at each time point).

Using our newly developed monarch-specific anti-CRY2 antibodies, the anatomical location of CRY2 staining by immunocytochemistry was very similar to the RNA expression pattern (Figure 6A). CRY2-like immunoreactivity was detected in the cytoplasm of ∼16 cells in the PI and four cells in the PL (Figure 6B and 6C). Double labeling studies showed that the CRY2 staining was localized in the PL to the four cells that co-express corazonin (Figure 6D and 6E) and TIM. Direct comparison confirmed co-localization of CRY2 and TIM in the same four cells in the PL (Figure S11). There were ∼25 CRY2-positive cells in the dorsal OL, ∼35 in the ventral OL, and ∼500 small CRY2-positive cells between the lobula and medulla and medulla and lamina. The main discrepancy between the RNA and protein patterns was that CRY2 staining was not detected in the RNA-expressing cells between the lamina and retina (Figure S10A versus Figure 6A). When the temporal profile of CRY2 staining in the PI, PL, and dorsal and ventral OL (the CRY2-positive cell groups in which signal intensity allowed for semiquantitative assessment) was analyzed over the circadian cycle, we found a significant circadian oscillation of cytoplasmic CRY2 staining in PL (*p <* 0.05), PI (*p <* 0.01), and OL (*p <* 0.01), which was most pronounced in OL (Figure 6F), with peak staining at CT 15.

Importantly, there was no detectable circadian oscillation in the ∼500 small cells in OL between lobula and medulla and between medulla and lamina, which compose over 90% of CRY2 staining in brain. This staining pattern accounts for our inability to detect a daily CRY2 oscillation in either head extracts (Figure 1B) or brains dissected away from photoreceptors (unpublished data). These CRY2-positive cells in OL overlap with those detected as expressing *cry2* RNA by in situ hybridization (Figure S10A); therefore, these cells in OL also likely account for the lack of a detectable *cry2* RNA rhythm in heads (Figure 1A).

### CRY2 Occurs in Nuclei of PL Cells at Appropriate Times to Repress Transcription

CRY2 nuclear staining should be observed in the PL at the time of transcriptional repression. Such evidence of nuclear translocation is expected based on the transcriptional feedback loop model of the *Drosophila* circadian clock [2] and on what we found for CRY2 in DpN1 cells (Figure 5E). Until now, we have not been able to find an obvious rhythmic nuclear accumulation of any clock protein so far examined (PER, TIM, CRY1, as well as CRY2) in the PL or in any other monarch brain region.

One possible explanation for not finding nuclear clock proteins is that each protein is heavily expressed in cytoplasm of PI and PL and, by comparison, there might be a relatively small amount of functionally relevant clock protein that does cycle into the nucleus to alter transcription, as appears to occur for phosphorylated nuclear PER bound to chromatin in *Drosophila* [28]. With this in mind, we initially examined CRY2 staining in thin (5 μm) sections throughout the entire monarch brain focusing on nuclear occurrence of CRY2 at 2-h intervals from ZT 18 to ZT 6, which covered seven points over the time interval in which we would expect to find CRY2 in the nucleus (Figure 6G and 6H), based on our studies of DpN1 cells (see Figure 5D and 5E). We compared the temporal pattern of nuclear CRY2 to the *per* RNA rhythm in monarch brain (Figure 6H, upper panel), because the *per* RNA rhythm is the most consistent clock gene rhythm in monarchs (Figure 1A), and it is the same assay we used as a transcriptional readout of dpCLK:dpCYC–mediated transcription for comparison with the temporal profile of nuclear CRY2 in DpN1 cells (Figure 5D, solid line).

In the PL, the nuclei are large (10 μm in diameter), and counterstaining with three specific fluorescent DNA probes revealed that these cells are unique in that most of the chromatin is distributed around the inner edge of the nuclear envelope and in small patches in the nucleus. In addition, the amount of DNA staining detected in the nucleus per se is minute, compared with nuclear staining in surrounding cells (Figure S12A). Nonetheless, using high-power microscopy in combination with a sensitive charge-coupled device (CCD) camera, we found clear evidence of temporal control of CRY2 staining in the nucleus of PL cells, which was limited to the four cells in PL and was not found in any other CRY2-positive cells in brain (Figure 6G and 6H). Specifically, over the 12-h period of study in LD, we identified nuclear CRY2 staining at ZT 2 and 4 only; no nuclear staining was detected at ZT 18, 20, 22, 24, or ZT 6 (Figure 6G, left column; Figure 6H, middle panel). The CRY2 nuclear staining in the PL co-localized with the chromatin detected in the nucleus by the DNA probes (Figure S12B). We next examined four time points over the circadian cycle and found CRY2 nuclear staining in PL only at CT 3, and not at CT 9, 15, or 21 (Figure 6G, right column; Figure 6H, lower panel). The timing of CRY2 nuclear occurrence correlated well with the time of maximal transcriptional repression of the *per* RNA oscillation in monarch brain (Figure 6H, upper panel), similar to the temporal profiles described in DpN1 cells (see Figure 5D and 5E). It is likely that CRY2 is present in the nucleus of relevant PL cells starting several hours before the peak, with the peak being what we are detecting for nuclear CRY2 in Figure 6G and 6H, based on our studies in DpN1 cells. We thus conclude that the cyclic presence of CRY2 in the nucleus of PL cells closes the circadian transcriptional feedback loop in vivo in the monarch butterfly.

We also looked at 5-μm sections for PER staining in the nuclei of PL cells over the circadian cycle using an antipeptide antibody that we previously used to characterize PER staining in monarch brain [13]. However, high background staining gave inconclusive results and no clear nuclear staining was detected above background at any of the Zeitgeber or clock times examined (unpublished data). Nonetheless, because of the strong evidence presented for CRY2 as a major transcriptional repressor of a clock feedback loop in monarchs (data in Figure 5), the detection of temporally controlled, nuclear CRY2 in putative clock neurons in butterfly brain helps resolve a puzzle that has existed for the last 10 y of work on lepidopteran clocks [26,30,31].

### CRY2-Positive Fibers Oscillate in the Central Complex

The site of the sun compass in insects now appears to be the central complex [18,19]. The central complex is a midline structure consisting of the dorsally positioned protocerebral bridge and the more ventrally situated central body, which has upper and lower subdivisions. Recent studies in locusts and *Drosophila* have shown that the central complex is not only a control center for motor coordination but is also the actual site of the sun compass (for polarized skylight integration from both eyes and probably all skylight information) [18], as well as being involved in visual pattern learning and recognition [32]. Finding a clock connection with the central complex in the monarch butterfly would be a major advance for beginning to understand its remarkable navigational capabilities.

**Figure 7:**
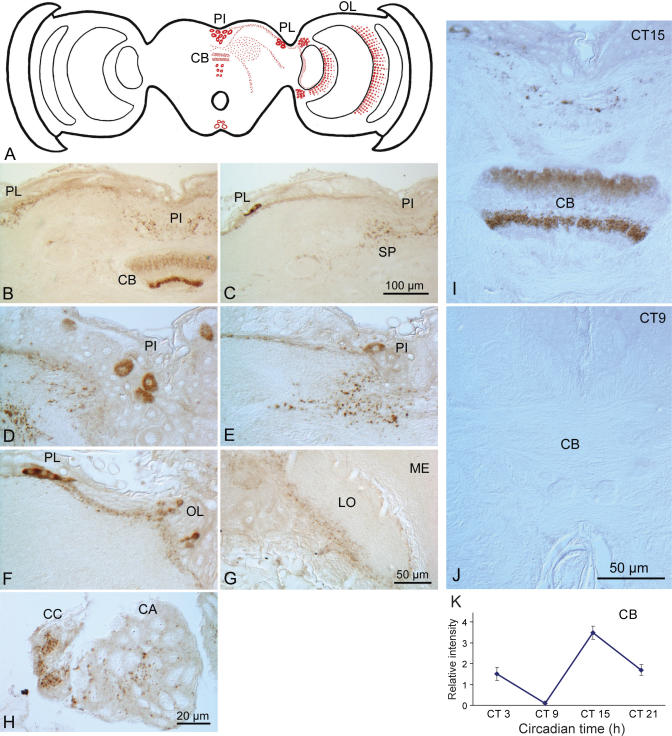
CRY2 Fiber Pathways in Monarch Brain (A) Schematic representation of frontal section illustrating the topology of CRY2 fibers at CT 15 using antibody CRY2-R42. A similar pattern of CRY2 fiber staining was found using antibody CRY2-GP51 (see Figure S13). PI, pars intercerebralis; PL, pars intercerebralis; OL, optic lobe; CB, central body. (B) CRY2 staining in central body (CB). PL, pars lateralis; PI, pars intercerebralis. (C–E) CRY2 fibers between PL and PI. SP, superior protocerebral bridge. CRY2 staining was not visible in central body on this section because the section is cut at a different plane. (F and G) CRY2 fibers between pars lateralis and optic lobe (OL); LO, lobula; ME, medulla. (H) CRY2 staining in corpora cardiaca (CC) and corpora allata (CA). (I and J) Circadian oscillation of CRY2 staining in the central complex. (I) CRY2 staining in upper and lower central body of the central complex at CT 15. (J) CRY2 staining in upper and lower central body of the central complex at CT 9. (K) Semiquantitative assessment of CRY2 staining in central body (CB) over the circadian day. Each value is mean ± SEM of five animals. Similar results were found in a replicate experiment using either CRY2-R42 or CRY2-GP51.

Both CRY2 arborizations and projections were identified in the brains of monarch butterflies (Figure 7A). The strongest and most dense arborization of CRY2 staining was found in the central body, just ventral from the protocerebral bridge (Figure 7B). This staining in the central complex was specific for CRY2, because staining for PER, TIM, or CRY1 was not detected in the central body. Another CRY2 arborization was found in the superior medial and lateral protocerebra, which are connected via the protocerebral bridge just above (dorsal to) the central body. In addition to these two arborizations, there are three CRY2-staining projections that could be traced. The first projection was coming from the protocerebral bridge and PI laterally toward the four cells in the PL (Figure 7C–7E). The second projection was extending from the superior lateral protocerebrum toward the OL (but it was not seen in the OL) (Figure 7F and 7G). The third projection traveled from the superior medial protocerebrum ventrally, likely to the corpora cardiaca/corpora allata complex, because CRY2 staining was detected in both these neurohemal organs (Figure 7H). It appeared that the CRY2 pathways arise from cells in PL and/or PI.

The CRY2-positive arborizations were under circadian control with strong staining in all areas at CT 15 and little to no staining detectable in those areas at CT 9. Dramatic CRY2 cycling was especially apparent in central body (Figure 7I–7K). These data provide evidence for a potential dual role for CRY2: as a core clock element and as an output that regulates circadian activity in the central complex.

### Conclusions

**Figure 8:**
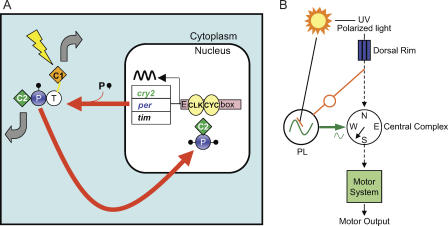
Proposed Monarch Butterfly Circadian Clock Mechanism and CRY-Centric Clock-Compass Models (A) The main gear of the clock mechanism in pars lateralis is an autoregulatory transcription feedback loop in which CLK and CYC heterodimers drive the transcription of the *per*, *tim*, and *cry2* genes through E box enhancer elements; in addition to *per*, there are CACGTG E box elements within the 1.5-kb 5′ flanking regions of the butterfly *tim* and *cry2* genes (unpublished data). TIM (T), PER (P), and CRY2 (C2) form complexes in the cytoplasm, and CRY2 is shuttled into the nucleus where it shuts down CLK:CYC–mediated transcription. PER is progressively phosphorylated and likely helps translocate CRY2 into nucleus. CRY1 (C1) is a circadian photoreceptor, which, upon light exposure (lightning bolt), causes TIM degradation to gain access to the central clock mechanism. The thick gray arrows represent output functions for CRY1 and for CRY2. (B) Clock-compass pathways in monarch butterfly brain. A circadian clock in the PL is entrained by light acting through CRY1 expressed in clock cells (orange line). A CRY1-positive fiber pathway (orange) connects the circadian clock to axons originating from polarized UV light-sensitive photoreceptors in the dorsal rim of the compound eye [13, 45]. The circadian clock also may interact directly with the sun compass (in the central complex) through a CRY2-positive fiber pathway (green) discovered in the current study. Output from the central complex ultimately controls motor output.

Collectively, our results provide several lines of evidence suggesting that monarch CRY1 functions in vivo as a circadian photoreceptor, whereas CRY2 functions as a transcriptional repressor for the butterfly clockwork. This novel clock mechanism has aspects of both the *Drosophila* and mouse circadian clocks rolled into one, as well as unique aspects of its own (Figure 8A).

The CRY1-TIM pathway for light-induced resetting of the monarch clock is similar to that found in the fruit fly, and the butterfly is the only other animal, outside of *Drosophila*, in which a photoreceptive function of CRY1 for clock entrainment has been shown in vivo. What is different between photoreceptive CRY function in fruit fly and monarch is that the cascade of protein degradation events ends with CRY2′s degradation in the butterfly, rather than with PER's, as occurs in *Drosophila*. We propose that it is the ultimate decrease in CRY2 levels that resets the CLK:CYC–driven transcriptional feedback loop in monarch butterflies (see temporal protein decay patterns in the light periods in Figure 1D).

Then what is the function of monarch PER? We have shown that PER is important for stabilizing CRY2, and PER:CRY2 heterodimers may also be involved in translocating CRY2 into the nucleus, as occurs in mammals [33], although we could not detect PER in the nucleus of PL cells using currently available antibodies. It is also still possible that PER has a minor role in repression of CLK:CYC–mediated transcription, although the dominant repressor in monarchs is CRY2.

The role of monarch CRY2 as a transcriptional repressor is similar to the role of the CRYs in the mouse clockwork [33]. The existence of CRY2 and its repressive function, independent of PER, are major distinguishing features of the monarch clock mechanism from that of *Drosophila*. *Drosophila* CRY has been suggested to function in the peripheral clockwork as a transcriptional repressor [34–36], but only when overexpressed with PER [37], and no such clock-like function driving behavior has been detected for fruit fly CRY overexpressed within the central clock of *Drosophila* [4]. We have been able to track monarch CRY2′s movement into the nuclei of PL cells at clock times appropriate for its role as a major transcriptional repressor of the butterfly clock feedback loop (Figure 6G and 6H)—no previous nuclear translocation of clock proteins has been reported in any other non-dipteran species. Our studies set the stage for more careful examination of this issue in other insects, as also suggested by a recent study in the housefly Musca domestica [38].

It is likely that monarch CRY2 exerts its inhibitory function on transcription by directly interacting with CLK:CYC heterodimers, which can now be assessed in DpN1 cells. DpN1 cells are also an important reagent for examining CRY1 signaling mechanisms, as it is the only insect cell line reported that has all the endogenous machinery from CRY1 light sensing through the degradation of CRY2.

The CRY-centric ancestral circadian clock we have defined in monarch butterflies may be common in those non-drosophilid invertebrates that express both *cry1* and *cry2*. The CRY-centric clock of the monarch may also hold a key to understanding the regulation of critical migratory behaviors, including time-compensated sun compass navigation [20,39,40]. The relatively intense staining of the CRY proteins in cytoplasm suggests output roles for the proteins distinct from those involved in the circadian clock mechanism and its entrainment by light (Figure 8A). Indeed, previous work has shown that a CRY1-staining neural pathway may connect the circadian clock to polarized light input entering brain that may ultimately impinge on the sun compass (Figure 8B; [13]).

The results presented here further show that a CRY2-staining neural pathway may more directly connect the circadian clock to the central complex (Figure 8B), the likely site of a sun compass [18,19], and that the pathway communicates circadian information to the sun compass (Figure 7I and 7J). CRY2 may simply be marking a circadian pathway to the sun compass or it may be directly involved in rhythmic synaptic activity in that region. The elucidation of a novel central clock mechanism in monarch butterflies and the finding of CRY-staining neural pathways to aspects of sun compass integration provide a solid cellular, molecular, and biochemical foundation for further functional and genetic studies into the remarkable navigational capabilities of the monarch butterfly.

## Methods

### Animals.

Monarch butterflies were purchased from commercial sources. The butterflies were housed in the laboratory in glassine envelopes in Percival incubators with controlled temperature (21 °C), humidity (70%), and lighting. The butterflies were fed 25% honey every third day.

### Cloning and sequence analysis.

cDNA fragments were cloned by degenerate PCR (see Table S1). cDNA templates for PCR were prepared from RNA purified from monarch butterfly whole heads or brains. The ends of the coding regions were obtained by rapid amplification of cDNA ends (RACE; Clontech kits). Complete open reading frames were obtained by PfuTurbo (Stratagene) PCR from cDNA. Clones were sequenced at core facilities at University of Massachusetts Medical School. Sequences were analyzed with MacVector (Accelrys) and the National Center for Biotechnology Information website (http://www.ncbi.nlm.nih.gov/BLAST/).

### Real-time PCR.

Total RNA was extracted using Trizol (Invitrogen). For head RNA extraction, an additional charcoal purification step was added before isopropanol precipitation to remove eye pigments and other factors that interfere with reverse transcription.

The quantifications of clock gene expression were done using real-time quantitative PCR by TaqMan probes with an ABI Prism 7000 SDS (Applied Biosystems). Total RNA was treated with RQ1 DNase (Promega), and random hexamers were used (Promega) to prime reverse transcription with Superscript II (Invitrogen), all according to manufacturers' instructions. PCR reactions were assembled by combining two master mixes. The first mix contained approximately 1 μg of cDNA template and 13 μl Platinum Quantitative PCR SuperMix-UDG w/ROX (Invitrogen) per reaction and was aliquoted into a PCR plate. The second mix contained forward and reverse primers (0.9 μM final concentration of each), probe (0.25 μM final concentration) and the water needed to bring each reaction to a final volume of 25 μl, and was subsequently aliquoted into the PCR plate.

The monarch *per* and control *rp49* primers and probes were identical to those reported previously [20]. The other primers and probes were as follows (F, forward primer; R, reverse primer; P, probe; all 5′-3′): monarch *timF*, CCAAACAGAGGACCAACAACAA; *timR*, CCTCGTTTGACGATCTTCTTTCTC; *timP*, FAM-TCGCGCTGGCGTAACGCTTCA-TAMRA; monarch *cry1F*, AAAGATGGTGGGCTACAATCGT; *cry1R*, CCTGAACTGCTGGTCCAAATC; *cry1P*, FAM-TGCGATACCTGCTGGAGGCGCT-TAMRA; monarch *cry2F*, CTGGAGCGACATTTGGAGAGA; *cry2R*, CAAGAGTGATTCTGGCGTCATCT; *cry2P*, FAM-AGGCTTGGGTCGCTTCGTTCGG-TAMRA. All primers and FAM-TAMRA labeled probes were purchased from Integrated DNA Technologies (Coralville). The efficiency of the amplification and detection by all primer and probe sets were validated by determin-ing the slope of Ct versus dilution plot on a 3 × 10^4^ dilution series. Individual reactions were used to quantify each RNA level in a given cDNA sample, and the average Ct from duplicated reactions within the same run was used for quantification. The data for each gene were normalized to *rp49* as an internal control and normalized to the average of all time points within a set for statistics.

### Insect cell culture, transfections, and transcription assays.

DpN1 cells were cultured in Grace's insect medium (Gibco 11605–094) supplemented with 10% fetal bovine serum (Gibco 26140–079). The cells were maintained at 28 °C in 25-cm^2^ plug seal flasks (Corning 430168) and split every 4 d.

The high-efficiency DpN1 cell expression vector, pBA, was derived from pIE/153A (V4+) vector (Cytostore), where the IE1 activator gene was removed by PCR. pBA-FLAG was generated by cloning the FLAG tag into the NotI site of the multiple cloning site of the vector. DpN1 expression plasmids that were used in luciferase reporter assays (Figure 5A) were generated by subcloning monarch *per*, *tim*, *cry1*, and *cry2* into pBA-FLAG, and monarch *clk* and *cyc* into pBA. The luciferase reporter *dpPer4Ep-Luc* was reported previously [16]. The normalization control was generated by subcloning β-galactosidase into the pBA vector. In addition, monarch *clk* and *cyc* were subcloned into a relatively low efficiency expression pIB5.1 vector (modified from the Invitrogen pIB vector, see [26]) to bolster the luciferase reading for experiments depicted in Figure 5B.

Transient transcription assays were done using 50 ng/well of *dpPer4Ep-Luc* as reporter and 50 ng/well pBA-*β-galactosidase* as normalization control. The cells were co-transfected with 50 ng/well of pBA-*clk*, pBA*-cyc*, and varying amounts of pBA-FLAG–*per*, –*tim*, –*cry1*, and –*cry2*. DpN1 cells were split into 12-well dishes and incubated at 28 °C for 2 d so the cultures were ∼50% confluent. Cells were then incubated in 300 μl serum-free Grace medium (Invitrogen) premixed with plasmids and 5 μl/well Cellfectin (Invitrogen) for 5 h. Grace's medium supplemented with 10% fetal bovine serum (700 μl) was added at the end of transfection. The cells were then incubated for 2 d before harvesting for luciferase assay, real-time quantification, and Western blot analysis.

For RNA interference (RNAi) experiments, dsRNAs were synthesized using the Megascript T7 transcription kit (Ambion) from PCR templates between 500–900 bp. Primers to generate PCR templates contain a T7 promoter at their 5′ ends, and the amplified regions correspond to cDNA locations in base pairs as: *GFP* (94–658), *per* (445-1374), *tim* (423–1,356), *cry1* (787–1,520), and *cry2* (311–924). 20 μg of dsRNA with 10 μl of Cellfectin per well were used to transfect 12-well culture dishes as above. For dsRNA treatment of DpN1 cells kept in LD, dsRNA and Cellfectin were incubated with the cells in serum-free Grace's media for 5 h during the light phase on the second day after the cells were split. At the end of transfection, serum-containing Grace's media was added to the dish. Cells were then incubated in an LD cycle for 2 d and harvested throughout the dark-to-light transition on day four.

### Antibody production.

We generated antibodies against monarch PER, TIM, and CRY2. Purified proteins containing the C-terminal 197 amino acids of PER, or amino acids 251–450 of TIM were used as immunogens in rats and guinea pigs [41]. For monarch CRY2, purified proteins containing the N-terminal 218 residues, the C-terminal 209 residues, or the full-length protein were used. Both affinity-purified and unpurified sera were used in Western blot, immunoprecipitation, and immunocytochemistry experiments.

Representative antisera to each clock protein were affinity purified and designed as follows: PER-GP40 (“GP” indicates raised in guinea pigs) for the antibody against PER; TIM-GP47 for TIM; and CRY2-GP51 and CRY2-R41 (“R” indicates raised in rats) for CRY2. The non–affinity purified antisera used included PER-R33, TIM-R38, CRY2-R42, and CRY2-GP50.

Specificity of the affinity-purified antibodies was evaluated by the size of immunoreactive bands as determined by Western blot of extracts from heads, brains, and DpN1 cells, compared with the exogenously expressed protein in S2 cells. Specificity was further verified by showing that the band intensity of endogenous protein was reduced by specific RNAi knockdown in DpN1 cells (see Figure 5B).

### Immunoprecipitation.

DpN1 cells were incubated in the dark for 4 d and homogenized in extraction buffer (20 mM HEPES, pH 7.9, 5% Glycerol, 100 mM KCl, 0.1% Triton X100, 1X Complete Protease Inhibitor [Roche]). Monarch brains were dissected from animals frozen at ZT 18. The photoreceptor layers of the eyes were removed, and the brains were then homogenized in the same extraction buffer. Insoluble cell debris was removed by centrifugation.

Protein G sepharose beads (GE Healthcare) were prepared for immunoprecipitation by washing three times in the extraction buffer. The beads were then incubated with rat anti-monarch PER (R33), TIM (R38), CRY2 (R41) antibodies for 1 h at room temperature. Normalized rat immunoglobulin G (IgG) (Santa Cruz Biotechnology) was used as control. The unbound antibodies were removed with an additional wash. Protein extracts were added to the beads and incubated overnight at 4 °C. The beads were washed three times with extraction buffer at 4 °C and then protein sample buffer was added. A Western blot was probed with guinea pig anti-PER (GP40), anti-TIM (GP47), and anti-CRY2 (GP51) antibodies.

### Immunocytochemistry.

Brain-suboesophageal ganglion complexes were dissected from CO_2_ anesthetized adult monarchs and processed immediately for immunocytochemistry as described earlier [30]. For examining nuclear localization of CRY2, the sections were counterstained with specific fluorescent DNA probes (DAPI, 1 μg/ml, 10 min at room temperature; Propidium iodide, 0.5 μg/ml, 10 min at room temperature; or YOYO-1 [Molecular Probes] 0.1 μM, 10 min at room temperature, respectively). Stained and mounted sections were examined using a Zeiss Axioplan 2 microscope equipped with Nomarski (DIC) optics, epifluorescence, and a CCD camera.

The following primary antibodies and their corresponding dilutions were used: TIM-R38 (1:500); TIM-GP47 (1:1,000); rabbit anti-CORAZONIN (from Makio Takeda, 1:1,000); CRY1-R31 (1:500); CRY1-GP37 (1:500); CRY2-R42 (1:200); CRY2-GP50 (1:200); and CRY2-GP51 (1:500). To visualize the primary antibody binding, the following secondary antibodies were used: horseradish peroxidase-conjugated goat anti-rat (1:1,000); horseradish peroxidase-conjugated goat anti-guinea pig (1:1,000, both from Jackson ImmunoResearch Laboratories); Alexa Fluor488-conjugated goat anti-rat (1:200); Alexa Fluor555-conjugated goat anti-rat (1:400); Alexa Fluor488-conjugated goat anti-guinea pig (1:200); Alexa Fluor555-conjugated goat anti-guinea pig (1:400), Alexa Fluor488-conjugated goat anti-rabbit (1:200); Alexa Fluor488-conjugated goat anti-mouse (1:200); Alexa Fluor555-conjugated goat anti-mouse (1:400, all from Molecular Probes).

To verify the specificity of immunological reactions, primary antibodies were replaced with normal goat serum. In an additional control of binding specificity, the anti-CORAZONIN antibody was pre-incubated with 100 molar excess of the original antigen prior to immunocytochemical staining. In all cases, no significant staining above background was observed.

For scoring of immunoreactive intensities, stained sections were coded and viewed under a microscope. Levels of staining were subjectively scored with an intensity scale from 0–5. The time of collection was decoded after scoring.

For immunocytochemistry of CRY2 location in DpN1 cells, cells were seeded on cover slips and entrained in LD at 28 °C for 2 d. The cells were fixed at the times indicated. The cellular localization of CRY2 was assayed by immunocytochemistry using anti-CRY2 (GP51) antibody and Alexa594 conjugated anti–guinea pig secondary antibody (Invitrogen). The cells were also stained with SYTOX Blue (Invitrogen) to visualize the nucleus.

### In situ hybridization.

For monarch *cry2*, the methods were similar to those above for immunocytochemistry except that after fixation in paraformaldehyde, the tissue was embedded in paraplast and sectioned (10 μm). In situ hybridization was carried out using the mRNA locator kit (Ambion). The riboprobes were localized by incubation with an alkaline phosphatase-conjugated anti-digoxigenin antibody (Boehringer and Mannheim; 1:500 dilution overnight at 4 °C), and visualized with BCIP and NBT (Perkin Elmer). DIG-labeled sense RNA probes were used in control experiments. In all cases, sense probes produced no signal.

### *Drosophila* studies.

For generating *UAS-cry1* transgenic lines, the 1,605-bp monarch *cry1* ORF was amplified from cDNA. To generate the untagged construct, the *cry1* product was cloned into the pUAST vector [42]. We created an N-terminal, *myc*-tagged monarch *cry1* construct by cloning the *cry1* PCR product into a myc-pUAST vector; the myc-pUAST vector was generated by cloning a BamHI-myc-BglII fragment, created using two oligos followed by primer extention, into the BglII site of pUAST. All constructs were sequenced. Both *cry1* constructs were injected into *y w*;*Ki p^p^[ry+Δ2–3]/+* embryos.

For generating *UAS-cry2* transgenic lines, the 2,229-bp monarch *cry2* ORF was amplified from cDNA. To generate the untagged construct, the *cry2* product was cloned into the pUAST vector. To generate the N-terminal, *myc*-tagged *cry2* construct, the *cry2* cDNA was cloned into the myc-pUAST vector*.* All clones were sequenced. Both *cry2* constructs were injected into *w^1118^* embryos by Genetic Services. During balancing, the *w^1118^* X chromosome was replaced with the y w-containing chromosome. Flies were reared and experiments were conducted at 25 °C.

For monarch transgene expression in *cry^b^* flies, the driver line was *tim-GAL4/CyO* [4]. The following lines were used: *cry^b^* (*y w*; *tim-GAL4/+*; *cry^b^*), *y w* (*y w*), *1a* (*y w*, *UAS-cry1#1a/Y*; *tim-GAL4/+*; *cry^b^ and y w*, *UAS-cry1#1a/y w*; *tim-GAL4/+*; *cry^b^*), *6b* (*y w*; *UAS-**myc**-cry1#6b/ tim-GAL4*; *cry^b^*), *15b* (*y w*; *UAS-* ***myc*** *-cry1#15b/ tim-GAL4*; *cry^b^*), *22b* (*y w*; *UAS-* ***myc*** *-cry1#22b/ tim-GAL4*; *cry^b^*), *19a* (*y w*; *UAS-cry2#19a/ tim-GAL4*; *cry^b^*), *18b* (*y w*; *UAS-* ***myc*** *-cry2#18b/ tim-GAL4*; *cry^b^*), and *125a* (*y w*; *UAS-* ***myc*** *-cry2#125a/ tim-GAL4*; *cry^b^*)*.*

For light pulse/phase shift experiments, 16 males per genotype per light pulse were entrained in 12:12 LD for three full days in 120–220 lux before receiving a 1-h light pulse at 1,000–1,400 lux (for CRY1 experiments) or 1,200–1,600 lux (for CRY2 experiments) at ZT 15 or ZT 21. [This small difference in light intensities between these two experiments was unfortunately unavoidable; we were unable to use the same incubator for both experiments, and there are enormous technical challenges in producing equivalent lux readings between incubators. Both of these experiments were performed at saturating light intensities and, thus, this difference should not affect the results.] A “no-pulse” control group was also included. Flies were then placed in DD for 6 d. Data were collected using the TriKinetics Drosophila Activity Monitor (DAM) system. To identify and exclude arrhythmic flies, 5 d of activity in DD were analyzed starting 12 h after the last “lights off” using the Fly Activity Analysis Suite (FaasX) CYCLE_P software (Michel Boudinot; michel.boudinot@iaf.cnrs-gif.fr) under the following parameters: no filter for high frequencies, chi-square significance 0.01. Matlab with the Signal Processing Toolbox and the FlyToolbox [43] was used to plot behavior peaks of pulsed versus nonpulsed flies. Phase shifts were determined for each genotype by taking the average delay or advance of the three peaks of activity after the light pulse. The first peak of activity directly after the light pulse was not included in the average.

For Western blot samples, eight males and eight females per sample were entrained in 12:12 LD for at least two full days before collecting on dry ice. Frozen fly heads were collected into Eppendorf tubes and homogenized in 30 μl lysis buffer [150 mM NaCl, 50 mM Tris-Cl, pH 7.4, 0.5% NP40, 100 mM NaF, Complete Protease Inhibitor Tablet (Roche)] with Kontes pestles. After centrifugation, 25 μl of the homogenate was transferred to fresh tubes. Protein concentrations were normalized by Coomassie Reagent (Pierce), and either 5 or 10 μg of protein was loaded per lane (depending on well size). Tubulin and dTIM/dpCRY2 were separated on the same gel and the filter cut at 75kDa. Primary antibodies were rat anti-dTIM (1:5,000) [6], and monoclonal mouse anti-Alpha Tubulin (Sigma) (1:8,000 or 1:16,000). Secondary antibodies (Santa Cruz) were goat anti-rat IgG HRP conjugated, goat anti-guinea pig IgG HRP conjugated, and goat anti-mouse IgG2a HRP conjugated. Films and chemiluminescent blots were imaged with the FUJIFILM LAS-1000, and bands were quantified using the ImageGauge V4.22 software.

## Supporting Information

Figure S1 PER Is Phosphorylated in Brains and DpN1 Cells Protein samples were prepared from extracts collected at ZT4. Phosphatase (800 units) was incubated with each protein sample at 30 °C for 30 min. After, the samples were immediately mixed with 2X SDS-PAGE loading buffer, boiled for 5 min, analyzed by Western blot, and probed with PER-GP40. Sodium vanadate was used to block phosphatase activity.
(97 KB PDF)

Figure S2 CRY1 and TIM Responses to Light after 48 h in DD (A) Clock protein abundance in DpN1 cells changes in response to light after prolonged exposure to dark. DpN1 cells were cultured for 48 h under DD and then exposed to light for 540 min. Cells were collected at the designated times. Cell homogenates were analyzed by Western blot and probed for CRY1 (GP37), TIM (GP47), PER (GP40), and CRY2 (GP51) (upper panel). The time courses of declines were quantitated by chemiluminescence, and band intensity was normalized against α-tubulin (lower panel). Time 0 is before lights on.
(B) Effects of inhibitors on the light-induced decrease in CRY1 and TIM. Cells were pretreated with DMSO (the vehicle control, left), MG115 (final concentration of 40 μM in DMSO) for 2 h prior to light exposure (center), or GSK-3β inhibitor VIII (final concentration 20 μM in DMSO) for 2 h prior to light exposure (right). CRY1 abundance (GP37) and TIM abundance (GP47) were monitored by probing Western blots of cells collected at the designated times during the 120-min light exposure.
(C) CRY1 mediates light-induced TIM degradation in DpN1 cells. Cells were pretreated with dsRNA against *GFP* (left), *cry1* (center), or *tim* (right) prior to light exposure. CRY1 abundance (GP37) and TIM abundance (GP47) were monitored by probing Western blots of cells collected at the designated times during the 120-min light exposure.
***Results:*** We found a light-induced decrease in CRY1 in untreated DpN1 cells after culturing the cells for 48 h in DD. Once lights were turned on after 48 h in DD, there was a rapid decrease in CRY1 and TIM, followed by a slower decrease in PER, followed by a decrease in CRY2(A), similar to the temporal cascade of protein decrements found in Figure 2. The delayed decrease in both PER and CRY2 abundance after light exposure was not due to accelerated protein synthesis, relative to CRY1 and TIM, because the same temporal sequence of declining protein accumulation was found following treatment of the cells with the protein synthesis inhibitor cycloheximide prior to light exposure (unpublished data).
The light-induced decrease in both CRY1 and TIM was blocked by the proteasome inhibitor MG115, showing that the decrease in CRY1 is mediated by proteosomal degradation (B, center), as occurs in *Drosophila* [10]. The lack of light-induced decrease in TIM with MG115 treatment was also likely due to lack of proteasomal degradation of TIM itself, as the decrease in TIM by light is not necessarily accompanied by a decrease in CRY1. In fact, the GSK-3β inhibitor VIII blocked the light-induced decrease in CRY1, but *did not* inhibit TIM's degradation by light (B, right). These data, along with the dsRNA data (C, center) show that CRY1 can mediate light-induced TIM degradation, with or without inducing its own degradation. The results further suggest the involvement of a GSK-3β-like kinase in the degradation of monarch CRY1 by light.
Using dsRNA, we showed that the light-induced decrease in TIM after 48 h in DD is also mediated through CRY1 (C). Pretreatment of cells with dsRNA targeting *cry1* prior to turning the lights on caused a substantial (70%) reduction in CRY1 in darkness just prior to light exposure (time 0) and greatly reduced the decrease in TIM abundance in response to light, compared to controls (cells treated with dsRNA against *GFP*). Double stranded RNA targeting *tim* reduced TIM abundance prior to and throughout light exposure, but did not deter CRY1′s rapid decrease following lights on. Collectively, the data show that CRY1 mediates the light-induced decrease in TIM in DpN1 cells, with or without inducing CRY1′s own degradation.
(375 KB PDF)

Figure S3 A Blue-Light Photoreceptor Entrains the Adult Eclosion Clock and Causes CRY1 and TIM Degradation in DpN1 Cells (A) Experimental paradigm for adult eclosion studies. Top panel shows the wavelength and relative light intensities used. Lower panel depicts the timing of the three light pulses (white, blue, and orange) during the dark period prior to placement in constant darkness. Pupae were kept in 12-h-light: 12-h-dark (LD) conditions for 7 d at 21 °C in a Percival incubator. The incubator was then put into constant dark (DD). During first night of DD, a 1-h light pulse was given at ZT 21 using a white light arc lamp (66901, Newport Oriel Instrument) with either an orange 540-nm long-wavelength pass filter (E540, Gentex) or a blue 450-nm broadband interference filter (57541, Newport Oriel Instrument). Light profiles were measured with a USB2000 spectrophotometer (Ocean Optics). Animal eclosion was monitored by standard video surveillance equipment. The number of animals eclosed per hour was recorded.
(B) Eclosion profiles for all four groups (including “no-pulse” control) for each of the 3 d in constant darkness.
(C) Data from all 3 d in DD for each group pooled relative to circadian time.
(D) Light effects on CRY1 and TIM degradation in DpN1 cells. After 48 h of culture in DD, cells were either kept in the dark or exposed to white light, blue light, or orange light, using the light filters described above. Cell homogenates were analyzed by Western blot and probed for CRY1 (GP37) and TIM (GP47).
(664 KB PDF)

Figure S4 Co-Localization of CRY1 and TIM in the PL Double-labeling immunofluorescence of CRY1 (using CRY1-GP37, left column) and TIM (using TIM-R38, right column) are shown for two different cells in the PL (upper and lower rows). Only two of the four TIM-positive cells in the PL co-localized with CRY1, which was found in 6/6 brains examined.
(695 KB PDF)

Figure S5 Distribution of TIM Immunoreactivity in Glomerular-Like Arborization and Adjacent Cells in the OL (A) Schematic representation of a frontal section illustrating the topology of TIM-immunoreactive cells using antibody TIM-R38. RE, retina; LA, lamina; ME, medulla; LO, lobula; PL, pars lateralis; PI pars intercerebralis.
(B–D) Double-labeling of TIM (B) and CRY1 (C, using CRY1-GP37) staining in the glomerular-like arborization/cells in optic lobe (arrow). D is the merged image.
(1.0 MB PDF)

Figure S6 A Light-Induced Decrease in CRY1 Is Not Essential for a Light-Induced Decrease in TIM in Either DpN1 Cells or Heads This was shown in LD by giving a 1- hr light pulse from ZT 14–15 or from ZT 20–21, monitoring clock protein levels at the end of each light pulse and 3 h later, and comparing the levels with cells and heads kept in darkness (Figure 2D); the formal properties of circadian clocks predict that light given early in the night (e.g., ZT 14–15) should delay the phase of the circadian clock oscillation, while light given late in the dark period (e.g., ZT 20–21) should advance the phase of the clock oscillation [44].
(A) Paradigm for light pulse study. Arrows indicate collection times.
(B and C) Effects of lights pulse on CRY1 (GP37) and TIM (GP47) levels in DpN1 cells (B) and heads (C). Protein levels were determined by Western blots. Band intensity was quantified by chemiluminescence, and the values were normalized against α-tubulin. For each timepoint, samples collected in the dark (gray bars) are plotted next to samples collected after a light pulse (red and blue bars). Each bar is the mean ± SEM of three experiments.
***Results:*** When a 1-h light pulse was given from ZT 14–15, TIM levels in both DpN1 cells and heads were significantly decreased, as expected, just after the light pulse (ZT 15), and the decrease was still present 3 hrs later (ZT 18) (B and C). However, there was no decrease in CRY1 abundance at either time point. Similar responses were seen in both DpN1 cells and heads when the light pulse was given from ZT 20–21 (B and C). In this instance, there was a small, but significant decrease in CRY1 3 h after lights off (ZT 0) in DpN1 cells (B).
(511 KB PDF)

Figure S7 Monarch PER Alone or in Combination with Submaximal Inhibitory Doses of CRY2 (A) or with TIM (B) Does Not Repress dpCLK:dpCYC–Mediated Transcription Using Luciferase Reporter Gene Assays The monarch butterfly *per* E box enhancer luciferase reporter (*dpPer4Ep-Luc*; 50 ng) was used in the presence (+) or absence (–) of monarch CLK/CYC expression plasmids (50 ng each). Monarch *cry2* (5 and 15 ng), *per* (5, 15, and 50 ng) or *tim* (5, 15, and 50 ng) was used. Luciferase activity relative to β-galactosidase activity was computed. Each value is the mean ± SEM of three independent transfections. Western blot of FLAG-epitope-tagged protein expression levels for each concentration of each construct is depicted below the graph in (A).
(500 KB PDF)

Figure S8 Effect of dsRNA against *per* on *cry2* RNA Levels DpN1 cells were treated with either dsRNA against *GFP* (ds *GFP*) or dsRNA against *per* (ds *per*)*.* PER and CRY2 levels were assessed by Western blot analysis, using PER-GP40 and CRY2-GP51 (upper panel). Blots were imaged by chemiluminescences, and band intensity was quantified. The results were normalized against α-tubulin. Corresponding RNA levels for *cry2* were assessed by qPCR (lower panel). The *cry2* RNA values are expressed relative to the value with ds *GFP* treatment (100%). Each value is the mean ± SEM of three experiments.
(141 KB PDF)

Figure S9 CRY2 Protein Levels in DpN1 Cells (A) Verification of specific knockdown of CRY2 in Figure 5D by dsRNA against *cry2* (lower blots) compared with CRY2 abundance when treated with dsRNA against *GFP* (upper blots) at two time points (ZT 4 and ZT 12) over the 24-h period of study. CRY2-GP51 was used.
(B) Subcellular location of CRY2 in DpN1 cells. Photomicrographs depict CRY2 in nucleus only (left column) and in both nucleus and cytoplasm (right column). Upper row, CRY2 staining (CRY2-GP51); middle row, nuclear staining with SYTOX Blue; lower row, merged images.
(262 KB PDF)

Figure S10 CRY2 RNA Distribution in Monarch Brain (A) Schematic representation of a frontal section illustrating the topology of CRY2 RNA expression. RE, retina; LA, lamina; ME, medulla; LO, lobula; PL, pars lateralis; PI pars intercerebralis, SOG, suboesophageal ganglion.
(B) CRY2 RNA staining in a group of neurosecretory cells in pars intercerebralis (PI).
(C) CRY2 RNA staining in cells in pars lateralis (PL).
(3.1 MB PDF)

Figure S11 Co-Localization of CRY2 and TIM in the PL Double-labeling immunofluorescence of CRY2 (using CRY2-R42, left column) and TIM (using TIM-GP47, right column) are shown for a cell in the PL at ZT 18 (upper), ZT 21 (middle), and ZT 0 (lower). All four CRY2-positive cells in the PL colocalized with TIM, which was found in 4/4 brains examined.
(923 KB PDF)

Figure S12 The Nuclei of PL Cells and CRY2 Staining (A) Photomicrograph of a region near the PL stained with the nuclear stain propidium iodide. Arrows denote patchy nuclear staining in two CRY2-positive cells (two arrows for each cell), whereas arrowheads denote intense nuclear staining in surrounding cells.
(B) Nuclear CRY2 is co-localized with chromatin in the PL. The section (5 μm) was taken from a brain collected at ZT4. The section was stained for CRY2 (CRY2-R42; left) and counterstained with propidium iodide (middle); the staining in cytoplasm is due to overexposure to amplify the low intensity of nuclear staining. The merged image (right) shows co-localization (arrows)
(1.6 MB PDF)

Figure S13 CRY2 Staining in Monarch Brain Using Antibodies R42 and GP51 (A and B) Double-labeling immunofluorescence of CRY2 staining in three cells in the PL using R42 (A) and GP51 (B). The fourth cell was out of the plane of section.
(C and D) Double-labeling immunofluorescence of CRY2 staining in a cell in the PI using R42 (C) and GP51 (D). All CRY2 positive cells in PI were co-localized with the two antibodies.
(E and F) CRY2 fluorescence in lower division of the central body (CB) using either R42 (E) or GP51 (F).
(G and H) CRY2 DAB staining in upper and lower subdivisions of the CB using either R42 (G) or GP51 (H).
(2.8 MB PDF)

Table S1 Monarch Clock Genes Expressed in DpN1 Cell Line (95 KB PDF)

Table S2 Degenerate Primer Sequences (85 KB PDF)

### Accession Numbers

The GenBank (http://www.ncbi.nlm.nih.gov/Genbank/) accession numbers for the monarch genes discussed in this article are *period* (AY237279), *timeless* (AY367059), *Clock* (AY364477), *cycle* (AY364478), *crytochrome1* (AY860425), *cryptochrome2* (DQ184682), *casein kinase II* α (EF554579), *casein kinase II* β (EF554578), *shaggy* (EF554581), *double-time* (EF554580), *vrille* (AY576272), *Pdp1 ɛ* (EF649714), and *slimb* (EF649713).

## Abbreviations

CT: circadian time
DD: constant darkness
dsRNA: double-stranded RNA
LD: 12 h light:12 h dark cycle
OL: optic lobe
PI: pars intercebralis
PL: pars lateralis
S2: Schneider 2
SOG: suboesophageal ganglion
ZT: zeitbeger time
