# Supplementary material for: Cryptochromes Define a Novel Circadian Clock Mechanism in Monarch Butterflies That May Underlie Sun Compass Navigation
Source: PLoS Biol. 2008 Jan 8;6(1):e4. doi: 10.1371/journal.pbio.0060004 (PMC2174970; doi:10.1371/journal.pbio.0060004)

Figure S3. A Blue-Light Photoreceptor Entrains the Adult Eclosion Clock and Causes CRY1 and TIM Degradation in DpN1 Cells

Adult Eclosion Behavior

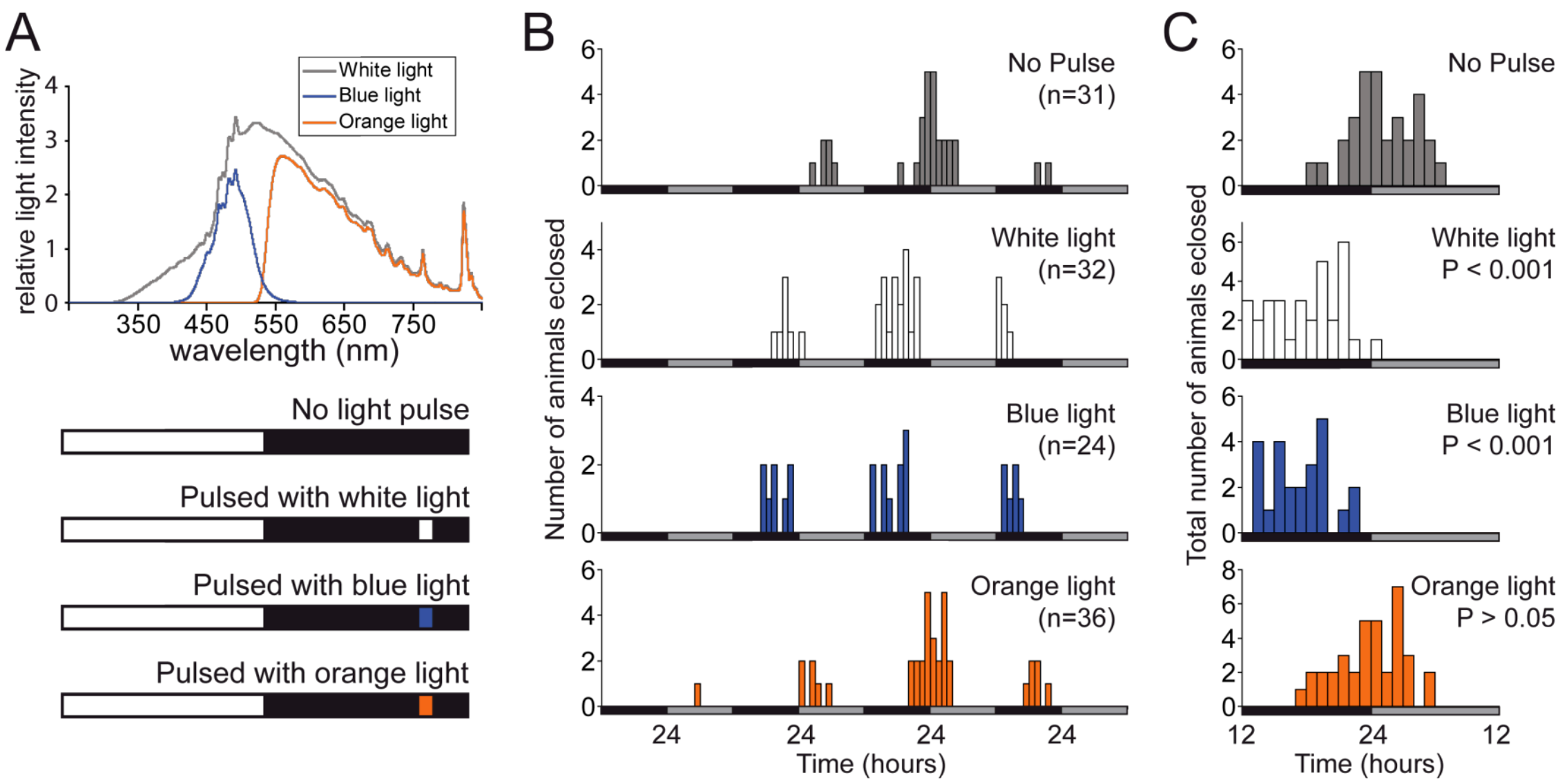

DpN1 Cells

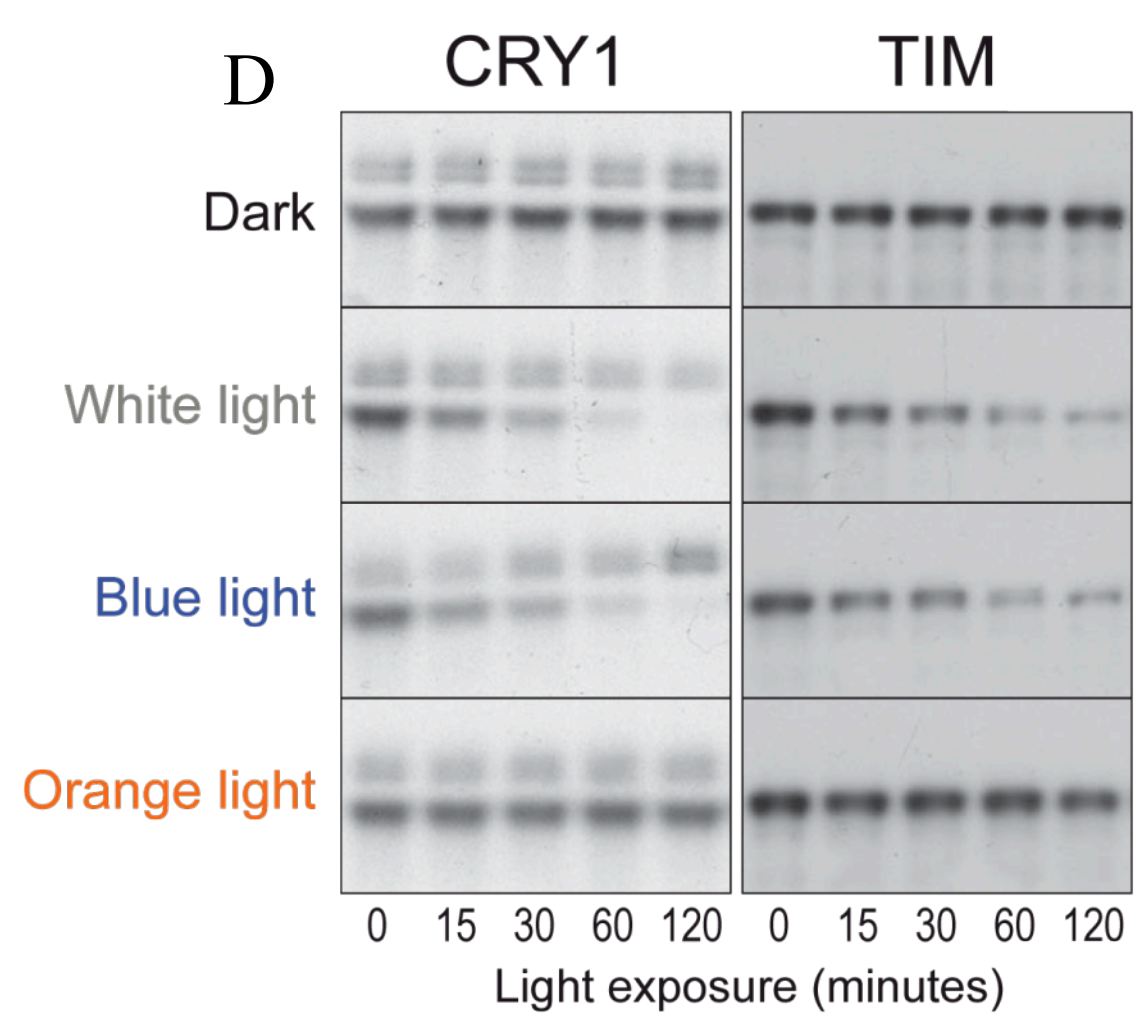

Supplement: Supplementary file 3 [file pbio.0060004.sg003.pdf]
