# Supplementary material for: Cryptochromes Define a Novel Circadian Clock Mechanism in Monarch Butterflies That May Underlie Sun Compass Navigation
Source: PLoS Biol. 2008 Jan 8;6(1):e4. doi: 10.1371/journal.pbio.0060004 (PMC2174970; doi:10.1371/journal.pbio.0060004)

Figure S7. Monarch PER Alone or in Combination With Submaximal Inhibitory Doses of CRY2 (A) or With TIM (B) Does Not Repress dpCLK:dpCYC-Mediated Transcription Using Luciferase Reporter Gene Assays

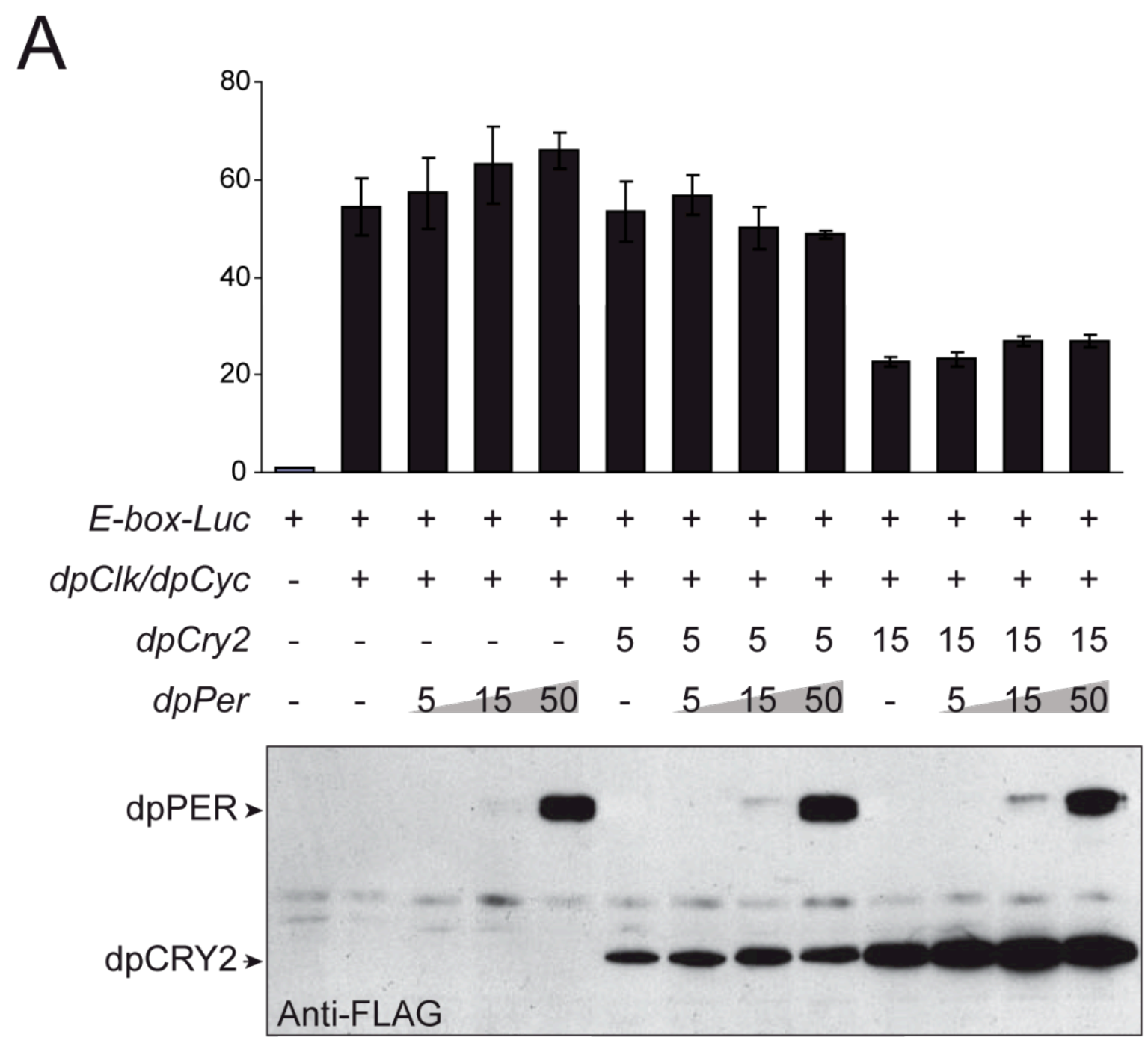

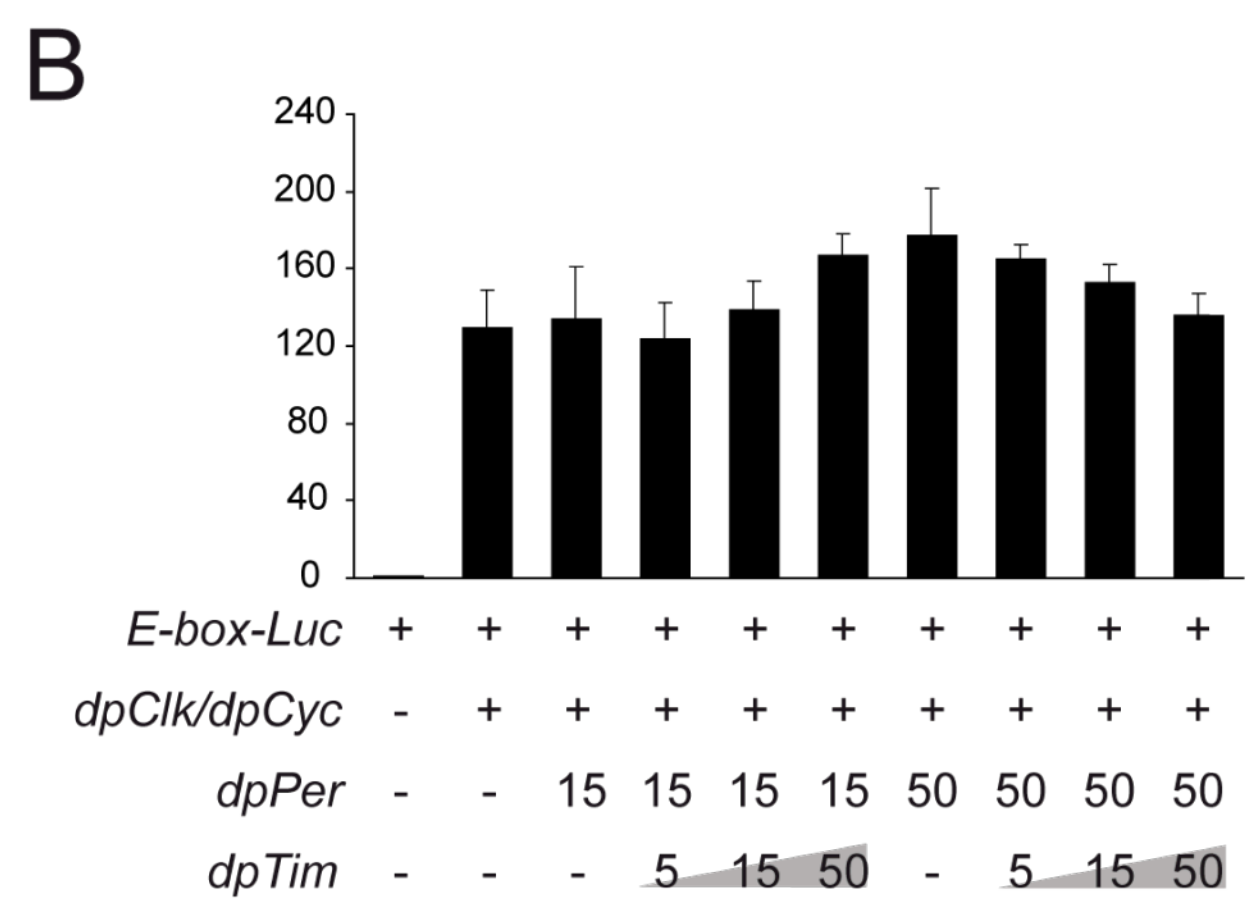

Supplement: Supplementary file 7 [file pbio.0060004.sg007.pdf]
