# Supplementary material for: Cryptochromes Define a Novel Circadian Clock Mechanism in Monarch Butterflies That May Underlie Sun Compass Navigation
Source: PLoS Biol. 2008 Jan 8;6(1):e4. doi: 10.1371/journal.pbio.0060004 (PMC2174970; doi:10.1371/journal.pbio.0060004)

Figure S10. CRY2 RNA Distribution in Monarch Brain

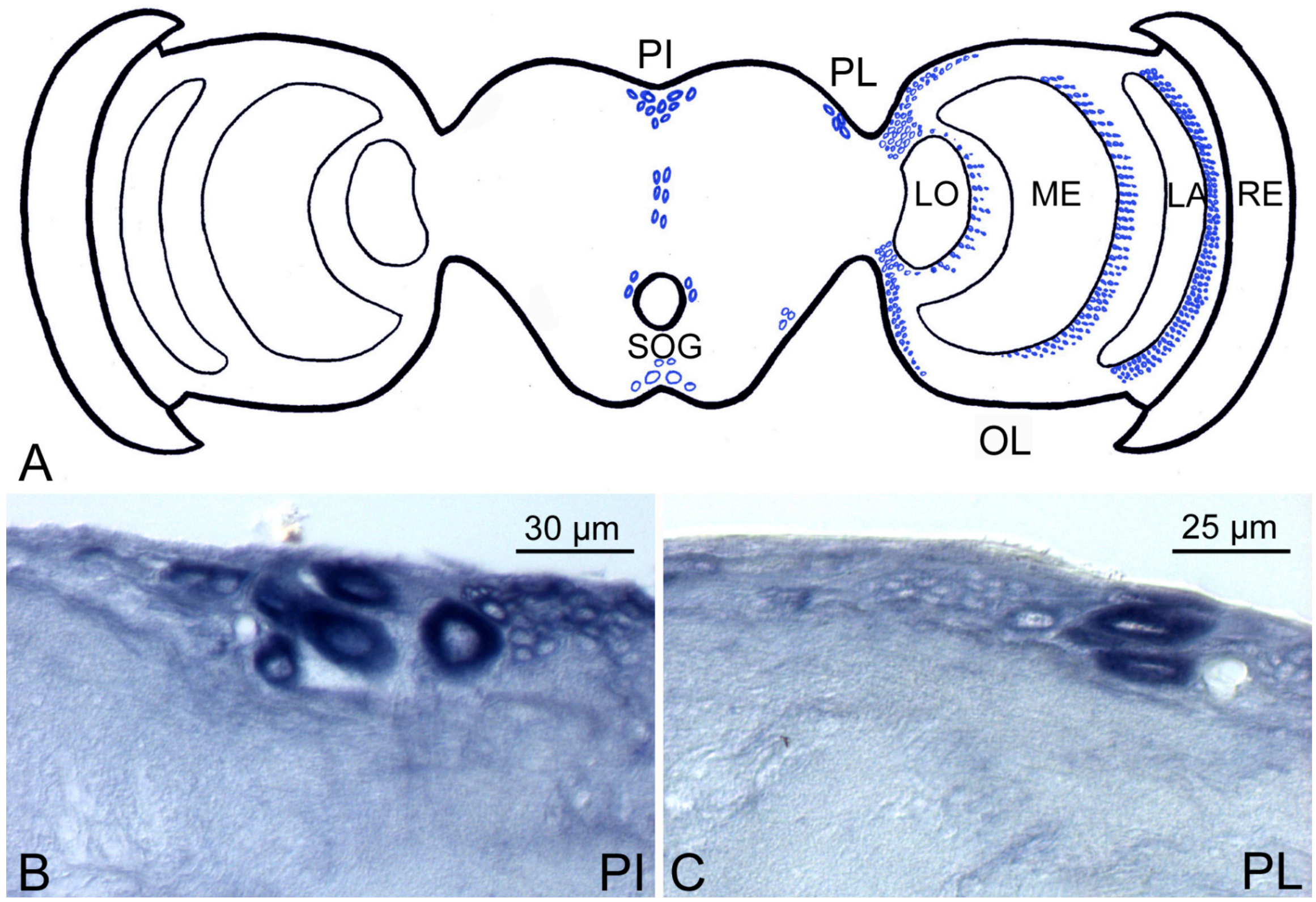

Supplement: Supplementary file 10 [file pbio.0060004.sg010.pdf]
