# Supplementary material for: Cryptochromes Define a Novel Circadian Clock Mechanism in Monarch Butterflies That May Underlie Sun Compass Navigation
Source: PLoS Biol. 2008 Jan 8;6(1):e4. doi: 10.1371/journal.pbio.0060004 (PMC2174970; doi:10.1371/journal.pbio.0060004)

Figure S11. Co-localization of CRY2 and TIM in Pars Lateralis

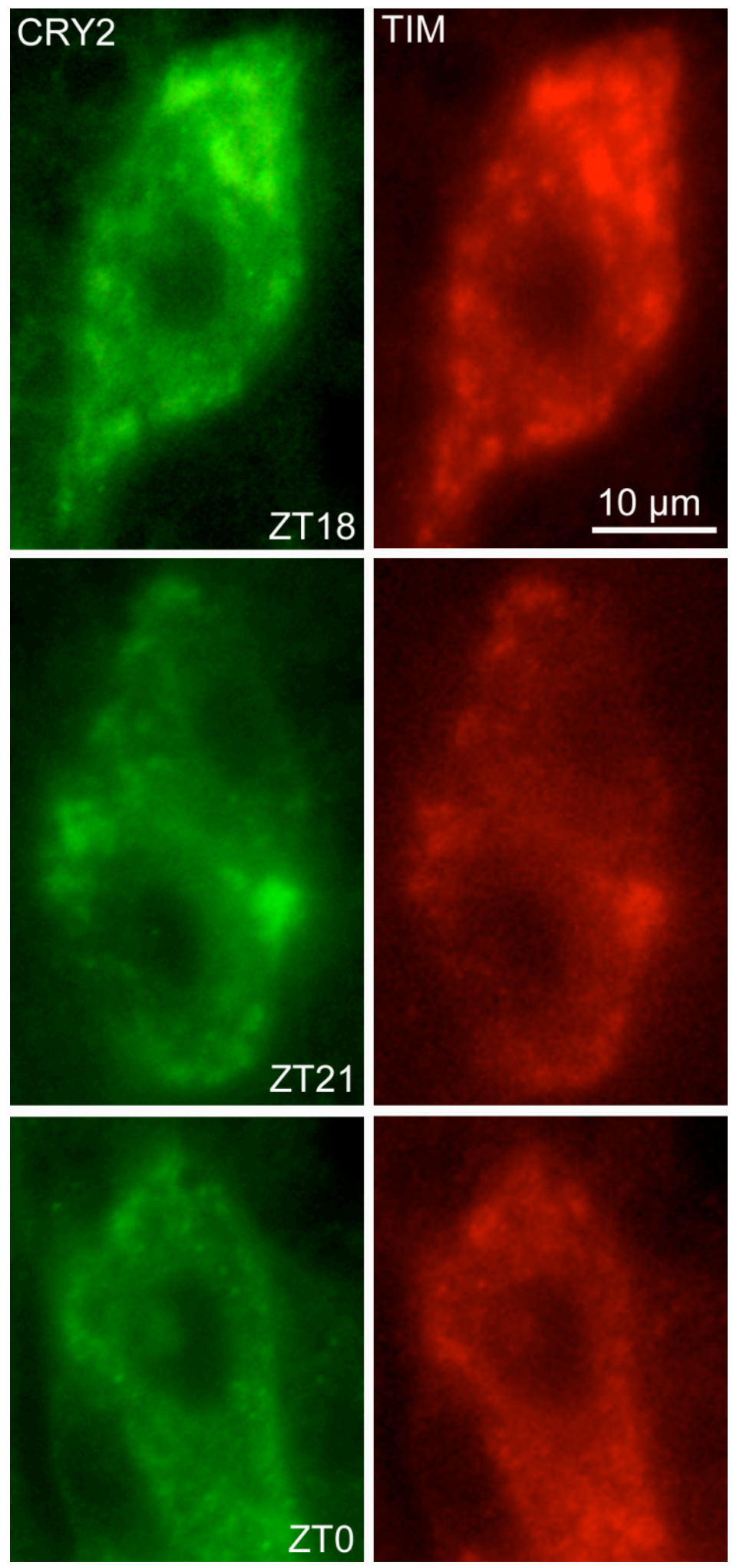

Supplement: Supplementary file 11 [file pbio.0060004.sg011.pdf]
