# Supplementary material for: Cryptochromes Define a Novel Circadian Clock Mechanism in Monarch Butterflies That May Underlie Sun Compass Navigation
Source: PLoS Biol. 2008 Jan 8;6(1):e4. doi: 10.1371/journal.pbio.0060004 (PMC2174970; doi:10.1371/journal.pbio.0060004)
Supplement: Supplementary file 14 [file pbio.0060004.st001.pdf]

Supplemental Table S1: Monarch Clock Genes Expressed in DpN1 Cell Line

**Gene**	**Accession #**	**Proposed Function in *Drosophila* clock**
period	AY237279	Clock gene
*timeless**	AY367059	Clock gene
Clock	AY364477	Transcription factor
cycle	AY364478	Transcription factor
cryptochrome1	AY860425	Circadian photoreceptor
cryptochrome2	DQ184682	N/A
*casein kinase II α**	EF554579	Phosphorylates PER
*casein kinase II β**	EF554578	Forms tetramer with alpha subunit ($\alpha_2\beta_2$)
*shaggy**	EF554581	Phosphorylates TIM
*double-time**	EF554580	Phosphorylates PER
*vrille**	AY576272	Represses *Clk* transcription
*Pdp1ε**	EF649714	Activates *Clk* transcription
*slimb**	EF649713	Ubiquitin-proteasome degradation of PER

Full-length open reading frame sequences have been submitted to GenBank except for *shaggy* and *Pdp1ε,* as only the N-terminal 407aa and the C-terminal 147aa have been cloned, respectively.

*Reported for the first time in this publication. Degenerate primer sequences are listed for each in **Table S2**.
