# Supplementary material for: Cryptochromes Define a Novel Circadian Clock Mechanism in Monarch Butterflies That May Underlie Sun Compass Navigation
Source: PLoS Biol. 2008 Jan 8;6(1):e4. doi: 10.1371/journal.pbio.0060004 (PMC2174970; doi:10.1371/journal.pbio.0060004)
Supplement: Supplementary file 15 [file pbio.0060004.st002.pdf]

Supplemental Table S2: Degenerate Primer Sequences

Gene	Primers	Position*
timeless	Forward 5'-ACNCCNACNGAYGAYGAYATHTC-3'<br>Reverse 5'-ATNACRTAYTCNAYNAGRTCNGACCA-3'	1360 bp<br>2213 bp
casein kinase II α	Forward 5'-GCCTTCTGGAGCTCGAGTGTAYRCNGAYGT-3'<br>Reverse 5'-CCAGAGCTTCAGGAGACACCARRTGYTGRTT-3'	42 bp<br>921 bp
casein kinase II β	Forward 5'-GAAGAAGTGTCTTGGATTACTTGGTTYTGYGG-3'<br>Reverse 5'-GGTCGTTTAGGTCGGTATTCAGGRTGNACCAT-3'	22 bp<br>544 bp
shaggy	Forward 5'-CGTGTCTTACATTTGCTCTCGATAYTAYMGNGC-3'<br>Reverse 5'GGGAATTTGAATTCAGTGTAGTTAGGRTTCATYTC-3'	740 bp<br>982 bp
double-time	Forward 5'-TGGAACTGCGAGTGGGTAAYAARTAYMG-3'<br>Reverse 5'-TTTGTCGTTTATTAGCAGCTTTCARNCCYTGCCA-3'	31 bp<br>699 bp
vrille	Forward 5'-CAACGAGAATTCATTCCAGACAAYAARAARGA-3'<br>Reverse 5'-TGTCGCAGTTTCAGTGGCARRCARTTRTT-3'	226 bp<br>806 bp
Pdp1 ε	Forward 5'-CTGACGAAGAACTGAAACCACARCCNATGAT-3'<br>Reverse 5'-CCACTTCTTGGTGCAGATTCATATTYTCYTTYTC-3'	131 bp<br>343 bp
slimb	Primary PCR:<br>Forward 5'-GGTGAACACTCTGATCCACCAYTGYGARGC-3'<br>Reverse 5'-GCCACCAGGTCCCACACYTTDATYTT-3'<br>Nested PCR:<br>Forward 5'-CGATTCAACAACGGTATGATGGTNAC-3'<br>Reverse 5'-AGGCAAGCACCGCATTCDATRTCCCA-3'	 834 bp<br>1313 bp<br><br>877 bp<br>1205 bp

* Position of the 5' end of each primer based on the monarch sequence submitted to GenBank.
